# Genome-wide association study identifies favorable SNP alleles and candidate genes for frost tolerance in pea

**DOI:** 10.1186/s12864-020-06928-w

**Published:** 2020-08-04

**Authors:** Sana Beji, Véronique Fontaine, Rosemonde Devaux, Martine Thomas, Sandra Silvia Negro, Nasser Bahrman, Mathieu Siol, Grégoire Aubert, Judith Burstin, Jean-Louis Hilbert, Bruno Delbreil, Isabelle Lejeune-Hénaut

**Affiliations:** 1 BioEcoAgro, INRAE, Univ. Liège, Univ. Lille, Univ. Picardie Jules Verne, 2, Chaussée Brunehaut, F-80203 Estrées-Mons, France; 2 GCIE-Picardie, INRAE, F-80203 Estrées-Mons, France; 3 GQE - Le Moulon, INRAE, Univ. Paris-Sud, CNRS, AgroParisTech, Univ. Paris-Saclay, F-91190 Gif-sur-Yvette, France; 4 Agroécologie, AgroSup Dijon, INRAE, Univ. Bourgogne, Univ. Bourgogne Franche-Comté, F-21000 Dijon, France

**Keywords:** Frost damages, Frost tolerance, Genome wide association study (GWAS), Pea (*Pisum sativum* L.), Quantitative trait loci (QTL), Haplotypes of markers, Candidate genes

## Abstract

**Background:**

Frost is a limiting abiotic stress for the winter pea crop (*Pisum sativum* L.) and identifying the genetic determinants of frost tolerance is a major issue to breed varieties for cold northern areas. Quantitative trait loci (QTLs) have previously been detected from bi-parental mapping populations, giving an overview of the genome regions governing this trait. The recent development of high-throughput genotyping tools for pea brings the opportunity to undertake genetic association studies in order to capture a higher allelic diversity within large collections of genetic resources as well as to refine the localization of the causal polymorphisms thanks to the high marker density. In this study, a genome-wide association study (GWAS) was performed using a set of 365 pea accessions. Phenotyping was carried out by scoring frost damages in the field and in controlled conditions. The association mapping collection was also genotyped using an Illumina Infinium® BeadChip, which allowed to collect data for 11,366 single nucleotide polymorphism (SNP) markers.

**Results:**

GWAS identified 62 SNPs significantly associated with frost tolerance and distributed over six of the seven pea linkage groups (LGs). These results confirmed 3 QTLs that were already mapped in multiple environments on LG III, V and VI with bi-parental populations. They also allowed to identify one locus, on LG II, which has not been detected yet and two loci, on LGs I and VII, which have formerly been detected in only one environment. Fifty candidate genes corresponding to annotated significant SNPs, or SNPs in strong linkage disequilibrium with the formers, were found to underlie the frost damage (FD)-related loci detected by GWAS. Additionally, the analyses allowed to define favorable haplotypes of markers for the FD-related loci and their corresponding accessions within the association mapping collection.

**Conclusions:**

This study led to identify FD-related loci as well as corresponding favorable haplotypes of markers and representative pea accessions that might to be used in winter pea breeding programs. Among the candidate genes highlighted at the identified FD-related loci, the results also encourage further attention to the presence of C-repeat Binding Factors (CBF) as potential genetic determinants of the frost tolerance locus on LG VI.

## Background

In 2018, the world area harvested of pea was ranking behind soybean, common bean, chick pea and cow pea, while the world production of pea was fourth to soybean, common bean and chick pea [1]. Average seed yield worldwide was about 1718 kg/ha in 2018, with the highest yields achieved in the Western European countries [1]. Dry peas are an important nutritional source which provide high quality protein for humans and for animal feeding [2]. In addition to the economic importance of pea seeds, pea crops have beneficial environmental impacts, mainly due to their ability to fix atmospheric nitrogen. They do not need nitrogen fertilizers and therefore help reducing N_2_O emissions [3]. For the past decades, spring sowing has been the most common method of cultivation for dry pea. However, the relatively short duration of the development cycle and various stresses such as biotic stresses, mainly *Aphanomyces* root rot and *Ascochyta* blight, as well as abiotic stresses, particularly hydric stress and high temperatures at the end of the development cycle, are at the origin of grain yield losses and variations [4]. Nowadays, winter peas are being developed in order to obtain higher and more stable yields. They are however limited by freezing temperatures during the winter time and the development of winter pea genotypes able to overcome freezing periods is thus desirable.

Mechanisms of tolerance to freezing temperatures have already been reviewed in many plant species. Plants can tolerate freezing temperatures using non-exclusive strategies: freezing escape and cold acclimation. Indeed, plants can escape freezing stress by delaying sensitive phenological stages, particularly floral initiation and flowering, given that frost sensitivity increases after floral initiation [5, 6]. Cold acclimation is the process by which certain plants increase their frost tolerance in response to low non freezing temperatures [7–9]. The *CBF/DREB* (*C-repeat Binding Factor/Dehydration Responsive Element Binding*) transcription factors have an important role in plant cold acclimation. These genes have been isolated first from *Arabidopsis thaliana* and belong to the *AP2/EREBP* (APETALA2/Ethylene-Responsive Element Binding Protein) family of transcription factors [10, 11]. In *Arabidopsis thaliana*, the CBF pathway is characterized by rapid cold induction of *CBF* genes by altering the expression of *CBF*-targeted genes, known as the CBF regulon, which in turn contribute to an increase in freezing tolerance [12]. Many studies have reported the significant role of *CBF* genes in freezing tolerance of herbaceous and woody plant species. Among these studies, the biological role of the CBF pathway for freezing tolerance has also been underlined by the colocalization of *CBF* genes with freezing tolerance quantitative trait loci (QTL) (Arabidopsis: [13], temperate cereals: [14–17], forage grasses: [18], legumes: [19]). Moreover, within the temperate cereals, *CBF* genes underlying FR2, a major homeologous frost tolerance QTL in barley, diploid and hexaploid wheat, are known to be structured in a cluster of tandemly duplicated genes [20]. In legumes a similar feature was found in *Medicago truncatula*, where a cluster of 12 tandemly duplicated *CBF* genes was shown to match with a major freezing tolerance QTL on chromosome 6 [19].

The identification of genomic regions controlling frost tolerance has initially been completed for cultivated species through the assessment of mapping populations and QTL mapping. In pea, QTL mapping studies for frost damages have been conducted in multiple field environments as well as in controlled conditions [21–23]. QTLs were detected using two populations of recombinant inbred lines (RILs), namely Pop2 and Pop9, respectively derived from crosses between Champagne (frost tolerant) and Térèse (frost sensitive) [21, 22] and China (frost tolerant) and Caméor (frost sensitive) [23]. Four common QTLs were detected within both populations. The corresponding regions were located on linkage groups (LG) III (two independent positions), V and VI. They explained altogether a major part of the phenotypic variation and were repeatable across environmental conditions. Three other loci were specific to one or other of the two populations and detected in fewer environments (Pop2: one locus on LG I, Pop9: one locus on LG VII, detected in one environment; Pop9: one locus on LG V, detected in two environments).

The resolution and accuracy with which QTL mapping can identify causal genetic determinism of the considered traits is limited by the high confidence intervals and the relatively low total number of recombination events in bi-parental mapping populations. In addition to QTL mapping, association studies have emerged as a complementary approach to dissect quantitative traits by exploiting natural genetic diversity and ancestral recombination events present in germplasm collections [24]. Used for more than two decades in human genetic research, association genetics have been adapted for genetic dissection in plants, taking advantage of the development of high-throughput genotyping resources in numerous species [24]. Genome-wide association studies (GWAS) aim at identifying genetic markers strongly associated with quantitative traits by using the linkage disequilibrium (LD) between candidate genes and markers. They rely on high-density genetic maps allowing an increased resolution of detection generating more precise QTL positions than bi-parental QTL mapping and give access to multiple allelic variation through the exploration of natural genetic diversity. In addition, GWAS can offer a powerful genomic tool for breeding plants by the identification of associated markers tightly linked to targeted genomic regions which can be used for marker-assisted selection. In the recent years, GWAS has been conducted in many plant species to dissect complex quantitative traits including winter survival and frost tolerance [25–33]. High throughput genotyping resources now available in pea [34] have also allowed to carry out GWAS in order to dissect the genetic determinism of resistance to *Aphanomyces euteiches*, plant architecture and frost tolerance. Desgroux et al. [35] studied associations for resistance to *A. euteiches* in 175 pea lines using a high-density SNP genotyping consisting of 13.2 K SNPs from the developed GenoPea Infinium® BeadChip [36]. Several markers significantly associated with resistance to *A. euteiches* harboring relevant putative candidate genes were identified. Significantly associated markers also allowed to refine the confidence interval of QTLs previously detected in bi-parental populations. Using the same SNP resource and a collection of 266 pea accessions, including the 175 former lines, Desgroux et al. also identified genomic intervals significantly associated with plant architecture and resistance to *A. euteiches*, of which 8 were overlapping for both traits [37]. In a different genetic background composed by a set of 672 pea accessions genotyped with 267 simple sequence repeat (SSR) markers, Liu et al. [38] detected 7 SSRs significantly associated with frost tolerance of which one was located on LG VI and was shown to colocalize with a gene involved in the metabolism of glycoproteins in response to chilling stress.

The present study aimed at reconsidering the regions of the genome that control frost tolerance in pea, taking advantage of genome-wide distributed SNPs generated from the 13.2 K GenoPea Infinium® BeadChip [36].

## Results

### Statistical analyses of phenotypic data

In order to undertake genome-wide association analyzes, a collection of 365 pea accessions, here after referred to as the association mapping collection or the collection, was phenotyped for frost damages (FD) under field and controlled conditions. Statistical analyses of frost damages scores showed highly significant genetic variation for all studied traits and the coefficient of genetic variation ranged between 37.6 for FD_Field_date4 and 74.4 for FD_Field_date2 (Table 1). The estimates of broad sense heritabilities (H^2^) were high for all traits, varying from 0.84 to 0.89. In addition, frost damages observed in controlled conditions and in the field for the date 3 and 4 showed the highest mean scores of 2.8, 2.4 and 2.4 respectively (Table 1). Frequency distributions of best linear unbiased predictions (BLUPs) values for each trait tended to fit normal curves within the collection (Additional file 1).

**Table 1:** Statistical parameters of the pea collection for the five observed traits

Trait	Number of accessions	Number of observations	Min	Max	Mean	SE	Vg	CVg	H^**2**^
**FD_CC**	363	1087	0.00	5.00	2.80	0.03	1.25	39.93	0.89
**FD_Field_date1**	363	1089	0.00	5.00	1.43	0.03	0.87	65.23	0.89
**FD_Field_date2**	363	1048	0.00	5.00	1.39	0.03	1.07	74.42	0.89
**FD_Field_date3**	363	1083	0.00	5.00	2.40	0.04	1.65	53.52	0.87
**FD_Field_date4**	363	849	0.00	5.00	2.41	0.03	0.82	37.57	0.84

Traits are abbreviated as follows: FD_CC: Frost damages in the controlled conditions experiment, FD_Field_date1, FD_Field_date2, FD_Field_date3 and FD_Field_date4: Frost damages in the field experiment at the first, second, third and fourth date of observation, respectively. Minimum (Min), maximum (Max) and mean values, standard error (SE), genetic variance (Vg), coefficient of variation of the genetic variance (CVg) expressed as √(Vg)/mean and broad-sense heritability (H^2^) are shown for each trait

### SNP genotyping of the association mapping collection

After quality control, the genotyping data comprised a total of 10,739 polymorphic SNPs with imputed missing data and a minor allele frequency (MAF) greater than 5%. Each linkage group contained 1533 SNPs on average. The distribution of SNPs varied within and between LGs (Additional files 2 and 3). Linkage group III showed the highest number of SNPs (1888 SNPs), while LG I was the least dense (1220 SNPs). Mean MAF in the association mapping collection varied from 0.29 on LGI to 0.31 on LG V and LG VI (Additional file 3). Only 751 of SNPs had a MAF less than or equal to 0.1 (Additional file 4). The distribution of SNP markers across the different LGs was dense and no gap between adjacent SNPs exceeded 1.7 cM, except on LG I and LG V which presented gaps of 2.3 cM (for the interval position 48.4–50.7 cM) and 3.7 cM (for the interval position 0.1–3.7 cM), respectively (Additional file 2). In addition, the map of the 10,739 SNPs used for GWAS showed an average number of 28 SNPs mapped at the same genetic position.

### LD analysis

The distribution of the estimate of the linkage disequilibrium (LD, *r*^2^) along to the genetic position for each linkage group as well as for the whole genome is presented in Additional file 5. The *r*^2^ value rapidly decreased as the genetic distance increased. The LD decay, estimated as the distance for which *r*^2^ decreases to half of its maximum level (0.22), was equal to 0.9 cM for the whole genome. Considering the LGs individually, the LD decay ranged from 0.3 cM for LG IV to 1.4 cM for LG V.

### Population structure and kinship analyses

**Fig. 1:**
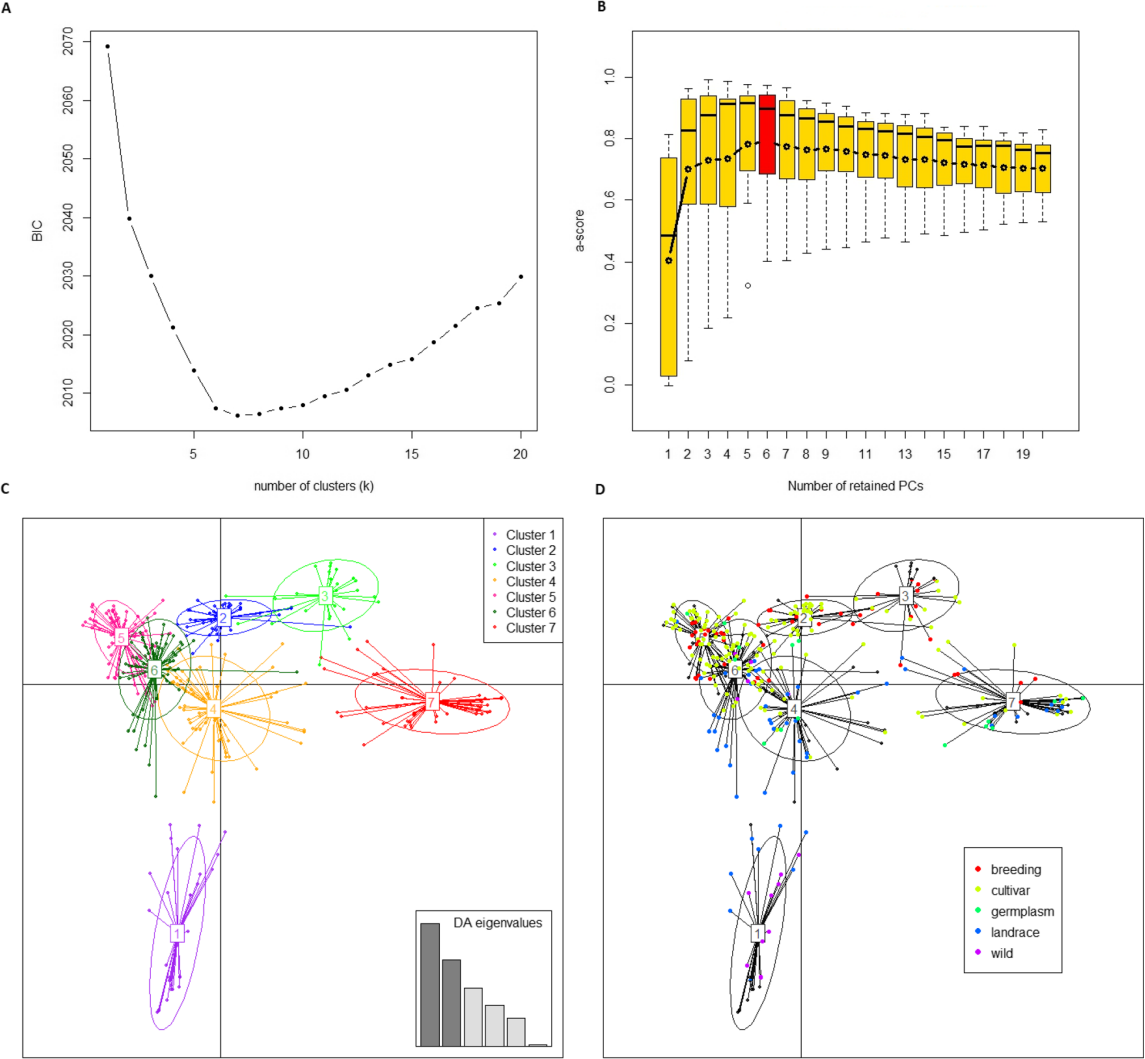
Population structure of the pea association mapping collection based on Discriminant Analysis of Principal Components (DAPC) analysis. **a** Number of clusters vs BIC values. The x-axis shows the potential numbers of clusters representing the population structure. The y-axis represents the BIC value associated with each number of clusters. **b** Number of principal components (PCs) vs a-score criterion. The x-axis shows the potential numbers of PCs which is used in the principal component analysis (PCA) step of DAPC. The y-axis gives the a-score criterion associated with each number of PCs. The optimal number of PCs, represented by a red bar, was obtained after 100 permutations. **c** Scatterplot showing the distribution of the association mapping collection along the first two principal components of the DAPC. Accessions are represented by dots and genetic clusters as inertia ellipses coded from 1 to 7. The bottom right inset shows eigenvalues of the six principal components in relative magnitude, ordered from 1 to 6 from the left to the right. **d** Scatterplot showing the correspondence between the classification of accessions for their cultivation status and the 7 clusters identified with DAPC; unknown accessions are shown by black dots

To avoid false positive results in association analysis, structure and kinship of the association mapping collection were analyzed using 2962 non-redundant positions markers. The collection structure was studied using the discriminant analysis of principal components (DAPC) method. Following the analysis of the Bayesian Information Criterion (BIC) profile and using the ‘a-score’ criterion, the optimal number of clusters was fixed to 7 (Fig. 1a) and the optimal number of principal components (PCs) was set to 6 (Fig. 1b). Therefore, these 6 PCs and 7 clusters were used for discriminant analysis of principal components. The distribution of individuals into the 7 clusters is represented along the two first axes of DAPC (Fig. 1c). The main passport data (Additional file 6) seemed to be related to the discrimination of the clusters. The cluster 1, comprising mainly wild peas and landraces from Africa and Middle or Far-East, was totally separated from the other clusters (Fig. 1d). The majority of accessions from clusters 2, 5, 6 and 4 were registered as spring sowing types while the winter sowing types were essentially gathered in clusters 3 and 7. These last two clusters differed for their end-use, the cluster 3 being mainly composed of field peas (81%) and the cluster 7 of fodder peas (80%).

The dendrogram using Nei genetic distance among accessions of the association mapping collection revealed also the presence of seven clusters (Additional file 7), as the DAPC method. For 79.61% of the accessions, the assignment to clusters performed by the dendrogram corresponded to the allocation made by the DAPC analysis (Additional file 6).

Within the kinship matrix (K), estimated for the whole genome, 85.7% of the kinship coefficient values were less than 0.1. 1.6% of these values were larger than 0.5. For the seven kinship matrices specific to each linkage group (K_LG_), the kinship coefficient values ranged similarly than for the K matrix (Additional file 8). These results indicated a weak relatedness between accessions and suggested that the majority of the accessions are genetically diverse, which was beneficial for subsequent GWAS mapping. From these results, the two first coordinates of DAPC results (Q matrix) and relatedness matrices (K matrix and K_LG_ matrix) were used as covariates for subsequent association analyses.

### Genome-wide association mapping

**Fig. 2:**
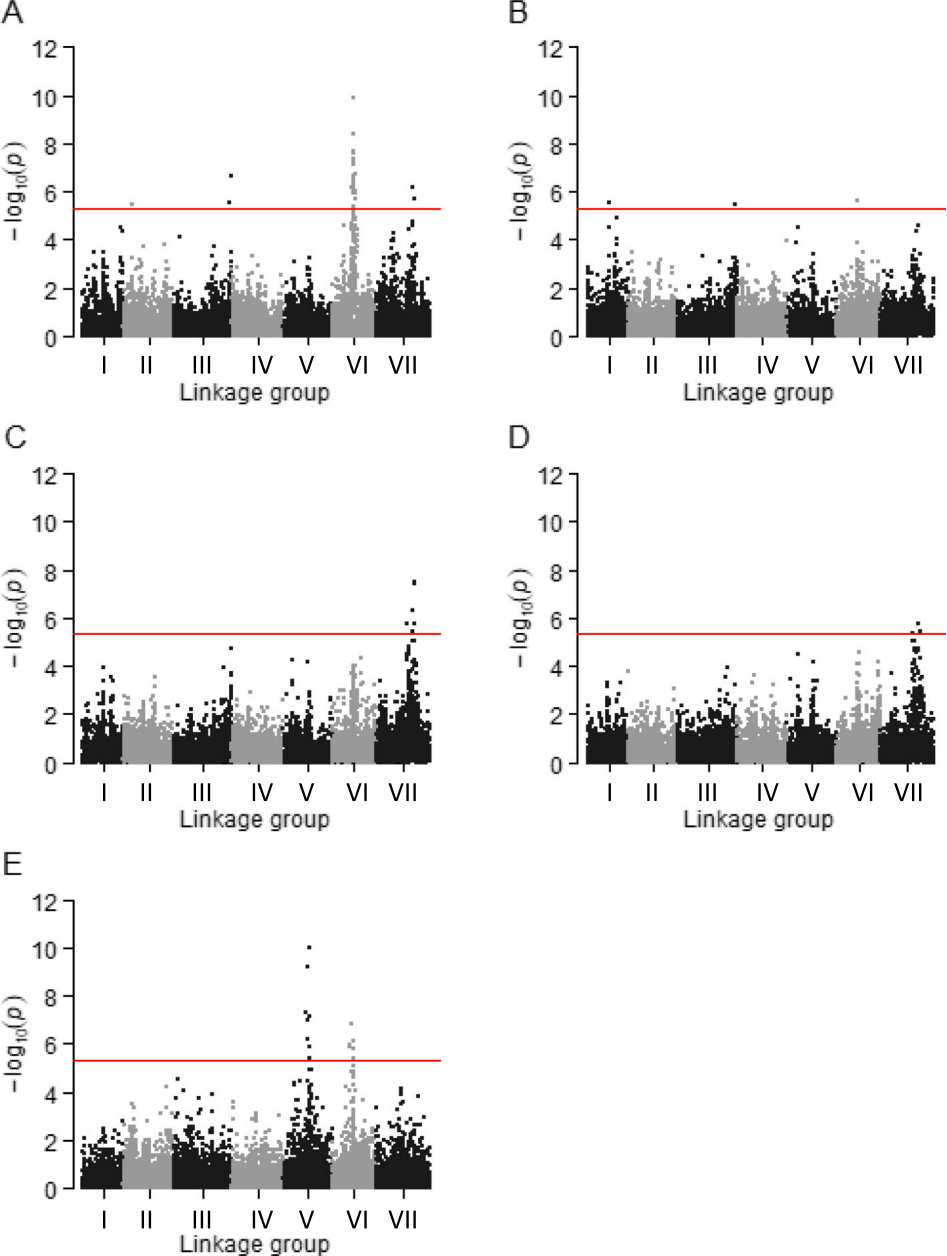
Manhattan plots of markers associated with the five frost damage traits. The plots show the *p*-values (*p*) for association between a phenotypical trait and each tested marker (expressed as the negative decimal logarithm of *p*, y-axis) plotted against the linkage group position of the marker (x-axis). Plots above the red horizontal line indicate the genome-wide significance with the Bonferroni threshold (−log10 (*p*) > 5.33). **a** is the plot for the evaluation of frost damages in the controlled conditions experiment. **b**, **c**, **d** and **e** are the plots for the four evaluations of FD in the field experiment, corresponding to the 4 dates of damages observation

**Fig. 3:**
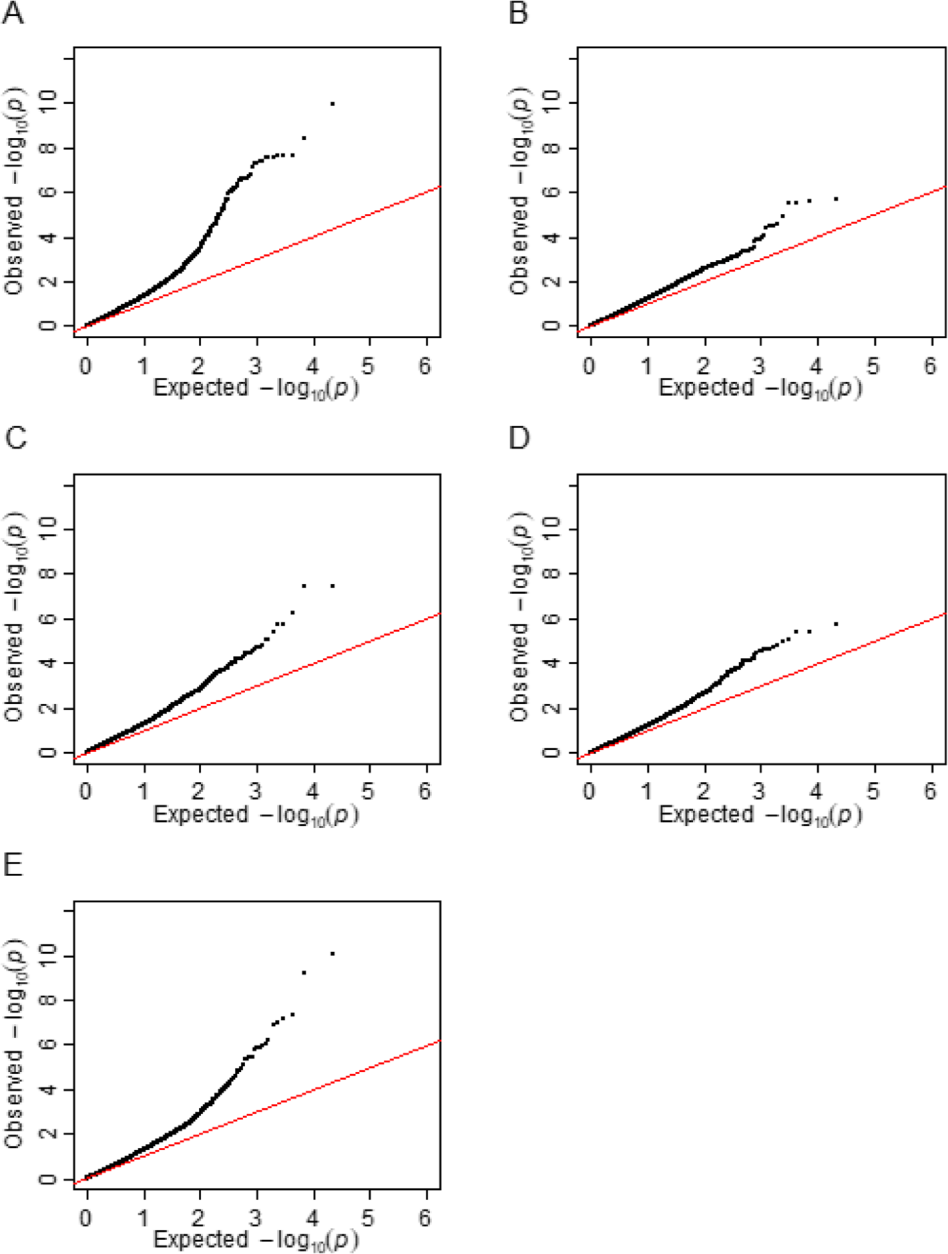
Quantile-quantile plots of the association mapping results. The plots show the observed p-values (*p*) for association between a phenotypical trait and each tested marker, expressed as -log10 of *p* (y-axis) plotted against -log10 of the expected p-values (x-axis) under the null hypothesis of no association for the analyses. **a** is the plot for the trait corresponding to the evaluation of frost damages (FD) in controlled conditions experiment. **b**, **c**, **d** and **e** are the plots for the four traits corresponding to the evaluations of FD in the field experiment

The comparison of BIC values of the four GWA-models tested, showed that the linear mixed model which included both Q and K_LG_ matrices as covariates was the optimal model for the following traits: FD_CC, FD_field_date1, FD_field_date2 and FD_field_date3. Whereas, the best fitting model for FD_field_date4 was the linear mixed model which included only K_LG_ matrices (Table 2). The Manhattan and their corresponding quantile-quantile plots of the association mapping results, run with the best model for each trait, are presented in Figs. 2 and 3. A total of 62 markers were significantly associated with any of the studied traits at the Bonferroni threshold -log10 (*p*) > 5.33. Frost Damage (FD)-associated markers were distributed on all linkage groups except LGIV. These SNPs exhibited a minor allele frequency ranging from 0.13 to 0.5. The number of markers associated with FD_CC, FD_field_date1, FD_field_date2, FD_field_date3 and FD_field_date4 were 40, 4, 6, 3 and 17, respectively. The highest *p* values were showed by the significant loci located respectively on LGV (9.67E-11) and LGVI (1.02E-10) (Table 3).

**Table 2:** BIC-based comparison of the four models used to control the rate of false positive associations

Trait	LMM1		LMM2		LMM3		LMM4	
	-2ln(L)	BIC	-2ln(L)	BIC	-2ln(L)	BIC	-2ln(L)	BIC
**FD_CC**	704.71	4073.94	701.16	4070.4	695.27	4064.51	691.68	**4060.92**
**FD_Field_date1**	658.87	4028.11	658.4	4027.63	675.69	4026.92	656.14	**4025.38**
**FD_Field_date2**	735.42	4076.81	732.06	4073.45	731.26	4072.65	728.85	**4070.24**
**FD_Field_date3**	837.83	4207.06	833.87	4203.1	836.8	4206.04	831.51	**4200.74**
**FD_Field_date4**	482.59	3489.84	481.45	**3488.7**	483.23	3490.48	482.53	3489.78

*LMM1* linear mixed model including the K kinship matrix, *LMM2* linear mixed model including the K_LG_ kinship matrix, *LMM3* linear mixed model including the K kinship matrix and the population structure matrix (Q), *LMM4* linear mixed model including the K_LG_ kinship matrix and the population structure matrix (Q). The model with the lowest BIC value (bold) is considered to be the best choice for the target trait

**Table 3:** Significant associations detected in the association mapping collection (363 accessions) for the five observed traits

Associated marker	Genetic map ^**a**^		Physical map ^**b**^		Associated trait	-log_**10**_ (***p***)	Minor allele (MAF) ^**c**^	Allelic effect ^**d**^	SE ^**e**^	Favorable allele	Allele of reference accessions			
	Linkage group	Genetic position (cM)	Chromosome	Physical position (bp)							Champagne	Térèse	China	Caméor
PsCam020765_11567_1428	I	47.5	2	160,946,678	FD_Field_date1	5.57	B (0.13)	0.18	0.04	A	AA	BB	AA	AA
PsCam043698_27686_2011	II	20.5	6	29,326,358	FD_CC	5.45	B (0.39)	0.18	0.04	A	AA	BB	AA	AA
PsCam049061_31705_537	III	131.1	5	566,158,491	FD_CC	5.55	B (0.50)	−0.29	0.06	B	AA	BB	AA	BB
PsCam035617_20792_637	III	132	5	566,953,569	FD_CC	6.63	A (0.50)	−0.37	0.07	A	BB	AA	BB	AA
					FD_Field_date1	5.47	A (0.50)	−0.31	0.07					
PsCam037922_22979_691	III	132.1	5	567,367,194	FD_CC	6.63	A (0.50)	−0.37	0.07	A	BB	AA	BB	AA
					FD_Field_date1	5.47	A (0.50)	−0.31	0.07					
PsCam051352_33858_733	V	53.6	scaffold01940	189,591	FD_Field_date4	7.29	A (0.25)	−0.27	0.05	A	AA	BB	BB	AA
PsCam048068_30823_2326	V	56.9	3	181,347,340	FD_Field_date4	6.21	B (0.33)	−0.26	0.05	B	BB	AA	BB	AA
PsCam002670_2123_708	V	58.6	3	184,294,454	FD_Field_date4	6.99	A (0.19)	−0.23	0.04	A	AA	BB	BB	BB
PsCam051635_34107_368	V	58.6	3	184,298,068	FD_Field_date4	9.21	A (0.27)	−0.33	0.05	A	AA	BB	BB	BB
PsCam011361_7735_182	V	60.2	3	189,446,769	FD_Field_date4	5.88	A (0.26)	−0.25	0.05	A	AA	BB	BB	BB
PsCam012913_8715_718	V	60.2	3	187,888,138	FD_Field_date4	5.43	A (0.32)	−0.22	0.05	A	AA	BB	AA	BB
PsCam049838_32453_1430	V	60.2	3	189,010,331	FD_Field_date4	7.17	B (0.28)	−0.26	0.05	B	BB	AA	BB	AA
PsCam042222_26315_1205	V	61	3	193,904,545	FD_Field_date4	10.01	A (0.24)	−0.38	0.06	A	AA	BB	BB	BB
PsCam021402_12023_752	VI	41.4	1	119,086,683	FD_Field_date4	5.84	A (0.21)	−0.23	0.05	A	AA	BB	BB	BB
PsCam043553_27552_265	VI	43	1	124,617,110	FD_Field_date4	5.95	A (0.22)	−0.25	0.05	A	AA	BB	BB	BB
PsCam022275_12527_210	VI	47.4	1	167,098,866	FD_CC	6.21	B (0.23)	−0.33	0.06	B	BB	AA	AA	AA
					FD_Field_date1	5.64	B (0.23)	−0.29	0.06					
					FD_Field_date4	6.86	B (0.23)	−0.27	0.05					
PsCam003541_2731_574	VI	48	1	165,049,951	FD_CC	6.51	A (0.44)	0.21	0.04	B	BB	BB	AA	BB
PsCam050834_33394_133	VI	48.5	1	165,743,845	FD_CC	5.35	A (0.33)	−0.25	0.05	A	AA	BB	AA	BB
PsCam001662_1375_798	VI	49.1	1	168,461,782	FD_CC	7.38	A (0.31)	−0.35	0.06	A	AA	BB	AA	BB
					FD_Field_date4	6.08	A (0.31)	−0.24	0.05					
PsCam043847_27818_181	VI	49.1	1	175,268,097	FD_CC	5.90	A (0.27)	−0.29	0.06	A	AA	BB	AA	BB
PsCam045419_29091_815	VI	49.1	1	169,979,594	FD_CC	7.57	B (0.37)	−0.24	0.04	B	BB	AA	BB	AA
PsCam048404_31138_2325	VI	49.1	1	170,956,086	FD_CC	6.02	B (0.31)	−0.30	0.06	B	BB	AA	BB	AA
PsCam004890_3696_1580	VI	49.1	1	181,902,508	FD_Field_date4	5.82	A (0.22)	−0.24	0.05	A	AA	BB	BB	BB
PsCam023246_13111_1125	VI	49.1	1	167,654,791	FD_Field_date4	5.40	B (0.21)	−0.25	0.05	B	BB	AA	AA	AA
PsCam037082_22189_1302	VI	49.1	1	181,965,322	FD_Field_date4	5.40	B (0.22)	−0.23	0.05	B	BB	AA	AA	AA
PsCam053556_35439_228	VI	49.1	1	166,361,064	FD_Field_date4	5.39	B (0.31)	−0.21	0.04	B	BB	AA	BB	AA
PsCam007060_5248_2156	VI	50.1	1	179,745,943	FD_CC	7.66	B (0.40)	0.22	0.04	A	AA	AA	AA	BB
PsCam026381_15234_450	VI	50.1	1	178,695,180	FD_CC	6.33	B (0.47)	0.21	0.04	A	AA	AA	BB	BB
PsCam037879_22937_948	VI	50.1	1	178,713,424	FD_CC	9.90	A (0.43)	0.26	0.04	B	BB	BB	BB	AA
PsCam001302_1089_1249	VI	50.2	1	182,819,711	FD_CC	6.50	B (0.46)	−0.22	0.04	B	BB	BB	AA	AA
PsCam003337_2589_220	VI	50.2	1	180,428,730	FD_CC	7.34	A (0.39)	0.22	0.04	B	BB	BB	BB	AA
PsCam037603_22679_2024	VI	50.2	1	180,042,543	FD_CC	7.59	A (0.38)	0.22	0.04	B	BB	BB	BB	AA
PsCam040539_25267_719	VI	50.2	1	181,440,108	FD_CC	7.59	A (0.38)	0.22	0.04	B	BB	BB	BB	AA
PsCam035398_20586_1094	VI	50.6	1	194,261,395	FD_CC	7.63	B (0.50)	−0.25	0.04	B	BB	AA	BB	AA
PsCam039054_24037_586	VI	50.6	1	210,266,228	FD_CC	6.30	B (0.41)	0.21	0.04	A	AA	AA	AA	BB
PsCam049925_32537_2703	VI	50.7	1	194,874,039	FD_CC	6.57	B (0.41)	0.22	0.04	A	AA	AA	AA	BB
PsCam038477_23510_469	VI	50.8	1	194,779,560	FD_CC	7.67	B (0.39)	−0.27	0.05	B	BB	AA	BB	AA
PsCam001148_968_692	VI	51.1	1	218,959,228	FD_CC	6.06	B (0.50)	0.22	0.04	A	AA	AA	BB	AA
PsCam006586_4888_245	VI	51.1	1	218,476,070	FD_CC	6.00	A (0.41)	0.20	0.04	B	BB	BB	AA	BB
PsCam017327_10745_93	VI	51.1	1	208,681,226	FD_CC	8.40	B (0.48)	−0.27	0.04	B	BB	BB	BB	AA
PsCam020872_11644_1756	VI	51.1	1	217,751,686	FD_CC	7.31	B (0.45)	0.23	0.04	A	AA	AA	BB	AA
PsCam025448_14564_2314	VI	51.1	1	218,939,803	FD_CC	6.08	A (0.29)	−0.22	0.04	A	AA	BB	BB	AA
PsCam033562_19160_307	VI	51.1	1	217,753,744	FD_CC	6.04	A (0.48)	0.21	0.04	B	BB	BB	AA	BB
PsCam036704_21832_970	VI	51.1	7	404,463,902	FD_CC	7.12	B (0.48)	0.22	0.04	A	AA	AA	BB	AA
PsCam038677_23691_325	VI	51.1	1	221,377,153	FD_CC	6.59	B (0.43)	0.20	0.04	A	AA	AA	BB	AA
PsCam044418_28295_579	VI	51.1	1	217,447,863	FD_CC	6.61	A (0.41)	0.21	0.04	B	BB	BB	AA	BB
PsCam052151_34533_410	VI	51.1	1	216,779,912	FD_CC	6.16	A (0.47)	0.21	0.04	B	BB	BB	AA	BB
PsCam053189_35238_569	VI	51.1	1	216,188,812	FD_CC	7.30	B (0.49)	0.23	0.04	A	AA	AA	BB	AA
PsCam042568_26621_42	VI	51.1	1	213,838,599	FD_Field_date4	5.43	A (0.21)	−0.25	0.05	A	AA	BB	BB	BB
PsCam035356_20546_778	VI	52.5	1	221,502,038	FD_CC	5.99	B (0.43)	0.20	0.04	A	AA	AA	BB	AA
PsCam040326_25099_1923	VI	52.5	1	226,457,822	FD_CC	5.91	A (0.38)	0.20	0.04	B	BB	BB	AA	BB
PsCam011774_8038_200	VI	52.8	1	232,110,795	FD_CC	6.76	A (0.49)	−0.23	0.04	A	AA	BB	BB	AA
PsCam023852_13541_264	VI	52.8	1	232,209,522	FD_CC	6.71	B (0.46)	−0.24	0.04	B	BB	AA	AA	BB
PsCam026055_15000_192	VI	52.8	1	228,344,153	FD_CC	5.70	B (0.44)	−0.22	0.04	B	BB	AA	AA	BB
PsCam001108_940_48	VII	72.9	7	338,172,762	FD_Field_date2	5.74	B (0.48)	0.20	0.04	A	BB	AA	AA	AA
					FD_Field_date3	5.34	B (0.48)	0.21	0.05					
PsCam050827_33387_242	VII	82.4	scaffold02790	184,253	FD_Field_date2	6.27	B (0.21)	0.21	0.04	A	AA	AA	AA	AA
PsCam037927_22984_97	VII	82.5	7	364,561,668	FD_Field_date2	5.40	B (0.41)	0.19	0.04	A	BB	AA	AA	BB
PsCam038378_23415_721	VII	86.4	7	416,077,744	FD_CC	6.18	A (0.46)	0.22	0.04	B	BB	BB	BB	AA
PsCam042427_26489_524	VII	87.1	7	417,288,899	FD_CC	5.67	B (0.41)	0.20	0.04	A	AA	AA	AA	BB
PsCam000301_265_1519	VII	87.9	7	424,418,432	FD_Field_date2	7.41	B (0.19)	0.23	0.04	A	AA	AA	AA	BB
					FD_Field_date3	5.72	B (0.19)	0.22	0.05					
PsCam045010_28743_3365	VII	88	scaffold02266	42,102	FD_Field_date2	5.73	B (0.21)	0.20	0.04	A	AA	AA	AA	BB
PsCam004928_3732_3087	VII	89.3	7	430,325,609	FD_Field_date2	7.46	B (0.28)	0.25	0.04	A	AA	AA	AA	AA
					FD_Field_date3	5.40	B (0.28)	0.24	0.05					

Traits are coded as described in Table 1. For each associated SNP, genetic and physical positions are indicated. ^a^: Linkage groups are named from I to VII and the genetic position is indicated in cM Haldane following the consensus map from Tayeh et al. [36]. ^b^: Physical marker position results from the projection of the marker onto the *Pisum sativum* v.1a genome JBrowse available at https://urgi.versailles.inra.fr/Species/Pisum [39]. Chromosomes are named in consecutive numerical order from 1 to 7 and physical positions are indicated in bp. Markers which are not assigned to one of the seven chromosomes are represented by their physical position on unanchored scaffolds. Significance threshold of the marker-trait association is -log_10_ (*p*) > 5.33 as described in the Material and Methods section. For each associated SNP, the allelic effect of the minor allele as well as the favorable allele are listed. ^c^: Minor allele frequency; ^d^: Allele effect of the minor allele. ^e^: standard error of the allelic effect. Alleles of four cultivars which are the progenitors of bi-parental mapping populations for frost tolerance are listed to illustrate contrasted genotypes for this trait: Champagne and China (frost tolerant), Térèse and Caméor (frost sensitive)

### Favorable alleles exploration for frost tolerance in pea

Twelve LD blocks were defined around the 62 significant FD-associated markers which included all markers in LD (*r*^2^ > 0.8) with the FD-associated markers (Additional file 9). FD-associated markers which were not in significant LD with any other SNP, and thus did not constitute a LD block, were also kept for further analysis. Finally, 75 SNPs, covering six FD-related loci distributed on LG I, II, III, V, VI and VII, were kept to identify favorable and unfavorable haplotypes for frost tolerance. For each of the six FD-related loci, marker haplotypes (two to nine) and corresponding representative accessions were identified (Additional file 10). For each locus, the effect of the different allele combinations was tested thanks to a variance analysis and a multiple comparison test of phenotypic mean effects (Additional file 10). These analyses identified 7 favorable haplotypes over the 6 FD-related regions carrying favorable alleles at each FD-associated marker except the haplotypes V.3 and VII.4 which contained each unfavorable allele, among 1 and 3 FD-associated markers respectively. Accessions carrying favorable haplotypes presented lower values of frost damages ranging between 0.00 ± 0.00 (haplotype VII.4 for the trait FD_Field_date2) and 2.65 ± 0.05 (haplotype III.1 for the trait FD_CC). Six groups of accessions carrying unfavorable haplotypes were also identified for which 100% of unfavorable alleles were observed over the significant FD-associated markers detected by GWAS. Typical accessions carrying favorable haplotypes and showing a mean score of frost damage ≤1 were mainly winter fodder peas (e.g. Black seeded, Champagne, Melrose, Blixt 7) (Additional files 6 and 10). While those carrying unfavorable haplotypes with a mean score of frost damage ≥4 were mainly spring garden peas (e.g. Automobile, Caroubel, Cennia, Ersling) (Additional files 6 and 10). The sequences of the 75 SNP markers related to frost tolerance trait are provided in Tayeh et al. [36] (Table S2).

### Candidate genes

The projection of the 75 FD-related markers on the pea genome assembly [39] allowed to define intervals of 2 Mb on all the pea chromosomes, except the chromosome 4 (LG IV). Four FD-related markers were assigned to unanchored scaffolds which were all less than 2 Mb long: in that case, annotated genes were listed for the whole scaffold. A particular case was encountered for the associated marker PsCam036704_21832_970 which is located on LGVI (51.1 cM) of the genetic consensus map [36] but which projection on the pea genome assembly locates on the chromosome 7 corresponding to the LG VII (Table 3). Considering this ambiguous position, we listed the gene corresponding to this marker as a unique candidate.

We located a total of 867 annotated genes, among which 277 corresponded to genes with unknown function according to the pea genome assembly v.1a (Additional file 11). Among the remaining 590 genes, we focused on gene families pointed as candidates in previous studies, i.e. *CBF*/*DREB* genes, genes coding for brassinosteroid receptors, genes implied in the production of gibberellin and genes implied in the synthesis of soluble sugars.

Nine candidates corresponding to, or at the vicinity of, FD-related markers were found to be annotated as AP2 domain genes in pea and this annotation could in some cases be refined thanks to the annotation of homologous genes in *M. truncatula* (Additional file 11). The marker PsCam037030_22140_221 on LG VI (49.1 cM), which is in high LD with all the significant FD-associated markers belonging to the LD block VI.2, belongs to the gene Psat1g103560 annotated as a *CBF* gene according to the annotation of the homologous gene of *M. truncatula* (Additional file 11). This FD-related marker was also annotated as a *CBF14* gene*,* following a blast search against *M. truncatula* carried out by Tayeh et al. [36]. Two other genes (Psat1g103560 and Psat1g103600) annotated as *CBF* genes were identified close to PsCam037030_22140_221, one of which being also precisely annotated as a *CBF14* by Tayeh et al. [36]. In the LD block VI.2, 2 FD-associated markers, namely PsCam023246_13111_1125 and PsCam007060_5248_2156, were also found to be close to AP2/ERF (Ethylene-responsive transcription factor) genes, namely Psat1g097280 and Psat1g097280, for which a precise study of the sequence is needed to verify if they belong to the CBF sub-family. Finally, 4 other potential candidate genes were found at the vicinity of the LD block VI.2, namely Psat1g103920, Psat1g106640, Psat1g115640 and Psat1g103680. These genes were annotated as *DREB* (Dehydration-responsive element-binding protein) genes, another term used to refer to *CBF* genes, in the homologous genes of *M. truncatula* (Additional file 11). These 4 genes lie in an interval of 35 Mb situated at a distance of 448 kb from the three other *CBF* genes mentioned above. One of them, Psat1g103920, contained the marker PsCam050192_32788_145 which was excluded from the GWA analysis because it showed a minor allele frequency lower than 0.05.

Three FD-associated markers, namely PsCam035617_20792_637 (LD-block III.1), PsCam048068_30823_2326 (LD-block V.1) and PsCam011774_8038_200 (LD-block VI.8) were found to correspond to 3 genes, namely Psat5g299600, Psat3g087400 and Psat1g119400 encoding brassinosteroid receptors (Additional file 11).

The FD-related marker PsCam037922_22979_691, which is only 0.1 cM apart from PsCam035617_20792_637 just mentioned above, was found to lie in Psat5g299720, a gene encoding for the gibberellin 3beta-hydroxylase enzyme and shown to correspond to the dwarfism gene *Le* in pea (Additional file 11).

Finally, three FD-associated markers belonging to the FD-related locus on LG VII, namely PsCam001108_940_48, PsCam037927_22984_97 and PsCam004928_3732_3087 were located within genes related to the synthesis of soluble sugars (Psat7g180280: endo-beta-1,3-glucanase, Psat7g193120: endo-1,4-beta-glucanase and Psat7g214880: beta-glucosidase G2, respectively) (Additional file 11).

## Discussion

### GWAS brings new insights into the determinism of frost tolerance in pea

In the present study, 75 markers associated with frost tolerance, 62 markers significantly detected by GWAS and 13 markers in high LD (*r*^2^ > 0.8) with one or the other of the 62 markers, were located among all the linkage groups of pea, except LG IV (Table 3, Additional file 9).

**Fig. 4:**
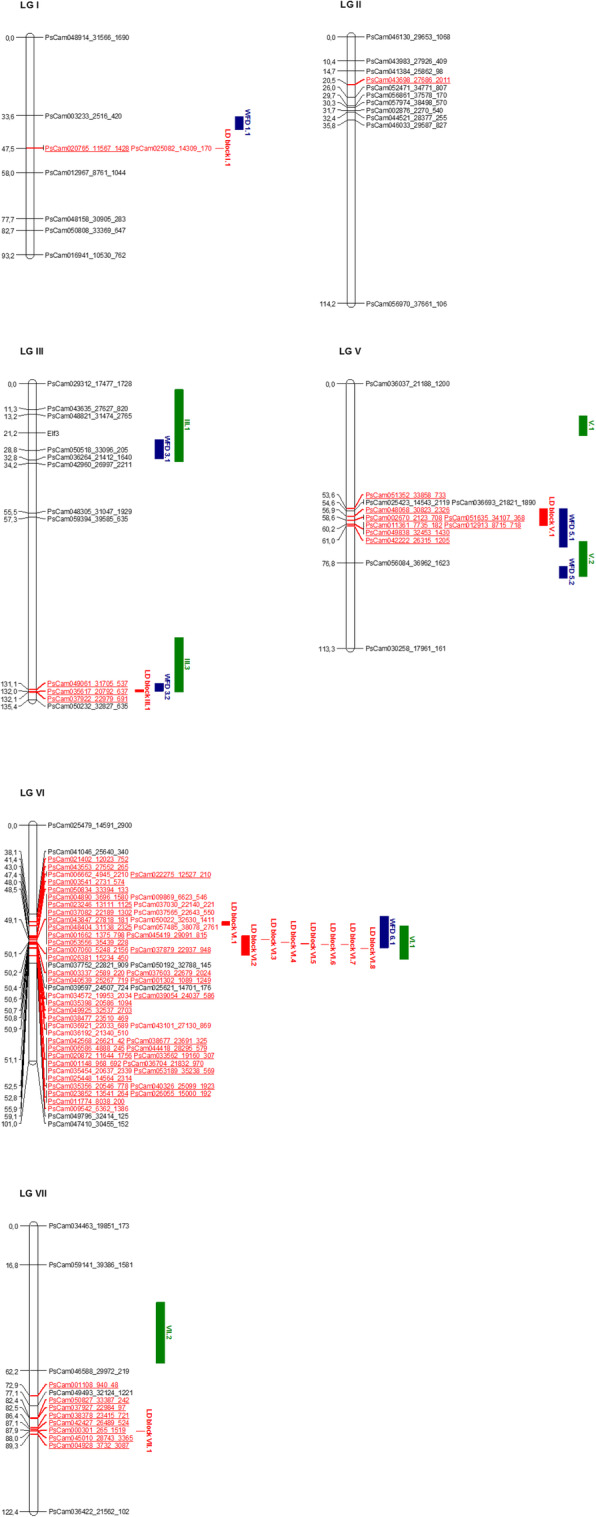
Comparative genetic map of genome-wide association study (GWAS) loci identified in the present study and quantitative trait loci (QTL) previously detected for frost tolerance in pea. Only linkage groups (LGs) with significant frost damages (FD)-associated markers detected by GWAS are presented. On each LG, SNP markers are shown on the right and genetic positions between markers are indicated in cM on the left. Frost tolerance loci detected in the present study are shown in red: FD-associated markers are in a red and underlined font; markers in linkage disequilibrium (LD; *r*^2^ > 0.8) with associated marker(s) are in a red and non-underlined font; the LD blocks identified by GWAS are drawn as red bars on the right of each LG. QTLs represented by blue and green bars were detected in the Champagne x Térèse [21, 22] and China x Caméor [23] populations respectively. For presentation purposes, only markers at the vicinity of significant loci and a few markers distributed along LGs are shown

Comparing the map positions with those of frost tolerance QTLs previously described, 3 regions corresponding to 62 markers among the 75 markers, were found to colocalize with 3 main QTLs previously detected by linkage mapping in two bi-parental populations for the same trait, namely WFD 3.2, WFD 5.1 and WFD 6.1 [21–23] (Fig. 4). These 3 QTLs were repeatedly detected in 5, 11 and 10 field conditions for WFD 3.2, WFD 5.1 and WFD 6.1, respectively. Moreover, the position corresponding to WFD 6.1 seems to also match with an EST marker recently found to be associated with frost tolerance in pea [38]: indeed, Liu et al. identified 7 marker-trait associations within a collection of 672 accessions, among which the marker EST1109 was located on LG VI within a functional gene that has a high homology with a gene encoding an alpha-mannosidase in *M. truncatula*. We reviewed the 1646 transcript-derived SNP markers mapped on LG VI by Tayeh et al. [36] and found that a marker corresponding to an alpha-mannosidase-like protein was located at 43.2 cM on the consensus map; consequently, as the FD-associated markers detected on LG VI in the present study are comprised between 41.4 and 52.8 cM, it is likely that the LG VI positions of both association studies coincide. Similarly, this study validated the QTL previously detected on LG III (WFD 3.2 in Pop2 [21], III.2 in Pop9 [23]) with a higher resolution than previous linkage mapping studies by the identifications of three main significant FD-associated markers, and whose positive alleles decreased the frost damage of accessions by an average of 0.33 (Table 3). All favorable alleles were carried by the sensitive genotypes Térèse and Caméor unlike the tolerant genotypes Champagne and China which had all undesirable alleles underlying this locus (Table 3). Altogether, the consistency of the 3 positions detected in both bi-parental mapping and association genetics reinforces their interest for breeding. The results presented here constitute an additional step towards the identification of underlying genes potentially involved in the control of frost tolerance, thanks to refined intervals provided by GWAS.

Unlike the correspondences with bi-parental mapping positions presented above, the present GWA study did not highlight any colocalization with a major QTL, namely WFD3.1, which was however found to be responsible for up to 52 and 19% of the winter frost damage variation within the RILs populations derived from Champagne x Térèse (Pop2) and China x Caméor (Pop9), respectively [21, 23]. The flowering gene *Hr* (*High response to photoperiod*), an orthologue of the Arabidopsis *Early flowering 3*, *Elf3* [40], was shown to be a relevant candidate for this QTL as it allows plants to be maintained in a vegetative state under short days and thus to escape the main winter freezing periods. It is likely that, in the case of the WFD3.1 position corresponding to the *Hr* gene, a strong correlation may have emerged between the population structure, possibly biased by the allelic variation at the *Hr* locus and the frost damage trait. This hypothesis relies on the observation that *Hr* may have been the target of natural selection for frost tolerance. Weller et al. [40] speculated that the *hr* mutation may have arisen within an ancestral pea lineage originating from the Near East domestication center and carrying the *Hr* allele. The *hr* mutation possibly permitted summer cropping in areas characterized by colder winters and is therefore highly represented in many domesticated lines of *Pisum sativum* at the origin of the current spring peas. To explore the hypothesis of undetection of a true association due to confounding with the population structure, we have verified the distribution of the *Hr* alleles, represented by their *Elf3* genotype, within the DAPC clusters of the association mapping population (Additional file 12). The *Hr* accessions, homozygous for the dominant allele *Hr*, are the main components of the clusters 7 (96%) and 1 (84%) and they represent 56.9% of the cluster 4. They are thus over represented in the three clusters gathering most of the winter sowing-type accessions, which may have contributed to a correlation between the frost tolerance trait and the population structure. The *hr* accessions, homozygous for the recessive allele *hr*, are the main components of the clusters 3 (100%), 2 (100%), 5 (98.7%) and 6 (95.7%), which are mostly spring sowing-type accessions. Consequently, we can suggest that the correction for the population structure (Q matrix) might have resulted in a structuring marker that probably cannot be detected by further association analysis using this sample of accessions. This kind of result has already been reported by Visioni et al. [27] who found that 2 of the 3 most significant SNPs of their study, tightly linked to major known genetic determinants of cold tolerance in barley, were undetected by GWAS if a correction by the structure was used. It was particularly the case for a SNP linked to *Vrn-H1*, a developmental locus governing barley vernalization requirement, which is for long a candidate for the frost tolerance locus *Fr-H1* but whose effect was suspected to be confounded with the population structure. To overcome this point, NAM-like linkage populations with bi-parental crosses in a reference design could be an interesting plant material for association mapping, in order to minimize population structure which may be necessary for dissecting the most structured traits.

Comparatively with consistent QTLs previously detected in bi-parental populations, the present GWA study also pinpointed three loci which have either not been detected yet (one region on LG II) or formerly detected in only one environment (two regions located on LG I and LG VII, respectively). The significant marker on LG II is supported only by one experiment in controlled conditions and must therefore be used with caution in breeding programs even if two distinct favorable and unfavorable haplotypes were identified (Additional file 10). The two markers significantly associated with frost tolerance on LG I are located at 47.5 cM on the consensus map, which lies in the projected confidence interval of a previous QTL, namely WFDcle.a, identified in one field condition with the *Hr* subpopulation extracted from Pop2 [22]. This colocalization slightly reinforces the consistency of this LG I position. In the same way, the 8 significant markers identified on LG VII with this study were located between 72.9 and 89.3 cM on the consensus map, which overlap with one former QTL, namely FD.c, which was detected once in a controlled chamber experiment [22], with the same *Hr* subpopulation. Thus, the colocalizing region on LG VII relies now on three independent experiments. In a panel of 672 accessions, Liu et al. [38] identified one marker on LG I and two markers on LG VII that were significantly associated with frost tolerance. It would be interesting to check the localization of these markers on the consensus map used here, as their position on the genetic map used by the authors [41] is not reported. Additionally, to these coincident positions for the frost tolerance trait on LG I and LG VII within different experimental conditions, the LG I and the LG VII regions detected in this study seems to also overlap with already detected QTLs for resistance to *Aphanomyces euteiches*. Indeed, both markers of the FD-related LD block I.1 lie in the confidence interval of the QTL *Ae-Ps1.1* identified by Hamon et al. [42], when the latter is projected on the consensus map used in the present work. Besides, the FD-related locus on LG VII includes the LD block VII.16 associated to the resistance to *Aphanomyces euteiches* [35], with which it shares the FD-associated marker PsCam038378_23415_721. The FD-related locus on LG VII deserves a particular attention for a further use in breeding for frost tolerance because accessions carrying the favorable haplotype underlying this locus (haplotype VII.4) shows very low scores of frost damages, ranging from 0 to 0.96, both in the field and controlled conditions experiments (Additional file 10). The relationships between haplotypes at this locus and the values for frost tolerance and *Aphanomyces euteiches* resistance will however have to be more precisely explored, to check if both traits can be bred favorably at this locus.

### GWAS detected frost tolerance-associated markers which are included in relevant putative candidate genes

The projection of the 75 FD-related markers on the pea genome assembly [39] allowed to identify 590 annotated genes with known putative protein functions, located in an interval of ±1 Mb on both sides of FD-related markers (Additional file 11). Among the diverse protein functions predicted, some are already related to the acquisition of frost tolerance in the literature.

Comparison of map positions has shown that the FD-related locus detected on LG VI in this study colocalizes with the previous QTL WFD 6.1, which is itself orthologous to a major QTL for frost tolerance in *M. truncatula* (Mt-FTQTL6) [43]. Tayeh et al. moreover showed that Mt-FTQTL6 covers a region containing a cluster of twelve *CBF* genes tandemly duplicated [19]. In the present study, 9 *AP2 domain* genes were found to correspond, or to be at the vicinity of, FD-related markers of LG VI. Among these genes, 7 are annotated as *CBF* or *DREB* genes. Given these results and the previous findings of Tayeh et al. concerning Mt-FTQTL6 [19], *CBF* genes located in the LD block VI.2, or at its vicinity, are also relevant candidates determining frost tolerance at this locus in pea. The potential role of *CBF* genes, and particularly *CBF14*, has already been highlighted in cereals. In wheat, Zhu et al. [44] showed that the natural variation for frost tolerance is mainly associated with a *frost resistance 2 (FR2)* locus including tandemly replicated *CBF* genes that regulates the expression of cold-regulated genes. Additionally, these authors proved that an increased copy number of *CBF14* was frequently associated with the tolerant haplotype of the locus *FR-A2* and with higher *CBF14* transcript levels in response to cold. Novák et al. [45] showed that *CBF14* genes contribute to enhance frost tolerance during cold acclimation in cereals.

Three candidate genes corresponding to FD-associated markers detected on LG III, LG V and LG VI, and annotated as brassinosteroid receptors, also appear in the literature to be implied in the crosstalk between plant hormone signaling in the cold stress response and the CBF regulon. In Arabidopsis, Eremina et al. [46] provided evidence that brassinosteroids contribute to the control of freezing tolerance. Indeed, these authors showed that brassinosteroid-deficient mutants of Arabidopsis were hypersensitive to freezing stress, whereas an activation of the brassinosteroid signaling pathway increased freezing tolerance both before and after cold acclimation. Furthermore, two brassinosteroids-responsive transcription factors have also been characterized as direct regulators of *CBF* expression through their binding to the promoters of these genes [46, 47]. In cultivated plants, the role of brassinosteroids is so far documented for the response to the chilling stress, as reviewed by Anwar et al. [48].

Within the FD-related locus of LG III, was identified a candidate gene encoding for the gibberellin 3beta-hydroxylase enzyme which produces bioactive gibberellin, also known as Le in pea (Additional file 11). Recessive *le* mutants at this locus are impaired in the production of gibberellin and produce a dwarf phenotype [49, 50]. In Arabidopsis, Achard et al. [51] undertook a molecular and genetic approach to evaluate the interaction between the *CBF1*-dependent cold acclimation pathway and the gibberellin pathway. They proposed a model in which the induction of *CBF1* expression by low temperature affects the gibberellin metabolism via upregulation of gibberellin-2-oxydase gene transcripts. The following reduction in bioactive gibberellin causes a higher accumulation of DELLAs, a family of nuclear growth-repressing proteins which in turns restrains plant growth. The *Le* gene has already been proposed as a candidate for the WFD3.2 and III.3 QTLs identified in the Pop2 and Pop9 populations, respectively. We considered the haplotypes of the parents of both populations at the corresponding FD-associated locus in this study and found that the favorable haplotype (III.1, including the dwarf allele at the *Le* gene) was borne by Térèse and Caméor, while the unfavorable haplotype (III.2, including the wild-type allele at the *Le* gene) was carried by Champagne and China. This observation is consistent with the favorable and unfavorable alleles determined by QTL mapping in bi-parental populations. As the three genes constituting the FD-related locus of LG III lie within a 1 cM interval, neither a synergistic effect nor linkage can be excluded.

Finally, three candidates underlying the FD-related locus on LG VII (chromosome 7) corresponded to genes related to the synthesis of soluble sugars. As accumulation of soluble sugars during cold acclimation is well documented in many plants, we can suggest that the LG VII-locus may have a role in frost tolerance by accumulating sugars in plant tissues during cold acclimation. Several roles for sugars in protecting cells from freezing injury have been proposed, including functioning as cryoprotectants for specific enzymes, as molecules promoting membrane stability and as osmolytes to prevent excessive dehydration during freezing, as reviewed by Xin and Browse [52].

### GWAS provides new markers and new genitors to breed for frost tolerance in pea

In the present study, 6 loci related to frost tolerance in pea were identified. At these 6 FD-related loci, 7 favorable haplotypes, carrying the highest number of favorable alleles at the FD-associated markers detected by GWAS, were significantly associated with the lowest scores of frost damages, i.e. the highest levels of frost tolerance. We identified 12 accessions showing lower scores of mean frost damages ranging from 0.13 to 1.04 and cumulating 6 (Glacier), 5 (Melrose, Blixt 7, Winterberger, Holly 9, Black seeded, Holly 17, Blixt 109, Fe and P1259) or 4 (Cote d’or and Picar) favorable haplotypes at the 6 FD-related loci (Additional file 13). All these accessions belong to the cluster 7 which was shown to be totally isolated from the other clusters by the DAPC analysis. They are fodder peas among which frost tolerant accessions have already been identified for breeding. The same 12 accessions also carry the *Hr* allele which was formerly shown to be favorable to frost tolerance [21]. One common point of these accessions except the accession named ‘Glacier, is that they carry the unfavorable haplotype at the FD-related locus on LG III comprising the *Le* gene. But rather than indicating a minor effect of the favorable haplotype at this locus, genetically linked to the dwarf *le* allele, this feature is to relate to the observation that field-autumn-sown *Hr* lines remain dwarf until a longer spring daylength has also triggered off the switch from the prostrate to the erected growth habit. This suggests an epistatic effect of *Hr* upon the expression of the dwarfism [21]. Comparatively to the above-described material, we also identified 16 accessions carrying all the unfavorable alleles at the FD-related loci located on LG V, VI and VII of the present study, additionally to the unfavorable allele *hr*. These accessions presented only 3 (Petit provencal, eM and Cador), 2 (Pi196033, Aldot, Chine-d368, 667, Merveille de Kelvedon, Miravil, Wav f502, Mingomark) or 1 (Ceia, Alaska, Finlande, Ersling and Automobile) favorable haplotype(s) identified on LG I, II and/or III, and which are mainly garden spring cultivars or breeding accessions presenting higher scores of mean frost damages ranging from 2.38 and 4.56. This allows us to state that the three loci on LG V, VI and VII play a bigger part in the frost tolerance on pea than the other FD-related loci located on LG I, II and III. Our results can help choosing tolerant progenitors and following favorable haplotypes through marker-assisted breeding. Furthermore, the FD-associated locus on LGV was found to overlap with the confidence interval of the frost tolerance QTL WFD 5.1 earlier detected within the Pop2 population [21] (Fig. 4). Comparatively to the linkage mapping method, with which a confidence interval of 16.6 cM was obtained, the GWA study enabled to refine the confidence interval to 7.4 cM (53.6 to 61 cM on the consensus map). This refined LG V locus presents a particular interest in breeding as it may provide markers to break the genetic relationship between the frost tolerance position and the neighbouring locus governing the seed trypsin activity. Trypsin inhibitors are known to be unfavorable for animal feed because they decrease the digestibility of protein [53]. The locus responsible for the seed trypsin activity (Tri) has been mapped on LG V [54] within the confidence interval of WFD 5.1 [21]. The favorable alleles at both loci are generally in repulsion. On the consensus map, this locus is represented by three markers, annotated as *trypsin inhibitor* genes [36] and located between 67.0 and 67.3 cM, 6 cM apart from the frost tolerance locus detected by GWAS. Thus, it seems possible to select favorable alleles for frost tolerance corresponding to the FD-associated markers detected by GWAS on LG V together with recessive alleles of the markers encoding for the trypsin inhibitor genes.

## Conclusion

In the present study, GWAS enabled to confirm QTLs significantly associated with frost tolerance such as WFD 3.2, WFD 5.1 and WFD 6.1. It also allowed to identify one region on LG II, which has not been detected yet and provided significant associations for two regions on LGI and LG VII that were formerly detected in only one environment. The results showed that GWAS is an effective strategy to identify markers precisely defining frost tolerance loci, which can be useful to breed for antagonistic traits as it is for the frost tolerance and Tri loci on LG V which are in linkage disequilibrium and in a repulsion phase. Our results also highlight that GWAS enables to find new sources of frost tolerance within collections of pea genetic resources. Finally, the present GWA study also brought to light the presence of CBF transcriptions factors as potential genetic determinants of the frost tolerance locus on LG VI, with one CBF-annotated marker being in high LD with significant FD-associated markers of the locus and six additional *CBF*/*DREB*-annotated genes mapped at the vicinity. As 12 tandemly duplicated *CBF* genes were already found to be relevant candidates underlying the orthologous frost tolerance QTL on *Medicago truncatula* chromosome 6, the hypothesis of a similar genomic organization in pea deserves to be tested.

## Methods

### Plant material

The association mapping collection, also named the collection, consists of 365 accessions (Additional file 6) from the pea reference collection described in Burstin et al. [55]. *Pisum* accessions from the collection represents a large genetic diversity ranging from wild peas (*Pisum fulvum*, *P. humile*, *P. elatius*, *P. speciosum*, *P. transcaucasicum* and *P. abyssinicum*) and landraces, to breeding lines and cultivars. This collection also represents a variability of genotypes based on the type of sowing (winter vs spring peas) and the type of end-use (fodder, field, mangetout, preserve and garden peas). Reference accessions which are the parents of bi-parental populations formerly used in QTL studies for frost tolerance [21–23], i.e. Champagne, China, Térèse and Caméor, are included in the collection.

All genotypes were purified for one generation by single seed descent (SSD) in insect-proof glasshouse. After this SSD generation, seeds were increased for one generation in insect-proof glasshouse. The seeds produced were sown in a nursery: tissue samples were harvested in bulk for DNA production from 10 sister plants and harvested seeds were used for phenotyping. When necessary, DNA was extracted again from the offspring of these plants. There is therefore zero or one generation between phenotyping and genotyping.

### Phenotyping

Frost tolerance of the collection was evaluated under field and controlled conditions. The field experiment was carried out at the INRAE (National Research Institute for Agriculture, Food and Environment) experimental station of Clermont-Ferrand Theix, France (45.72 °N latitude and 3.02 °E longitude at an altitude of 890 m) during the growing season of 2007–2008. Sowing date was 09 October 2007 and the date of emergence was 26 October 2007. Plots were sown in a randomized complete block design with three replicates. Weeds and diseases were controlled chemically.

The record of temperatures indicated that cold acclimation and freezing periods occurred during the experiment (Additional file 14). The collection was assessed for frost tolerance by visual estimation of winter frost damages after the main winter freezing periods have passed. As described in previous studies [21–23], a score was assigned to a plot as a whole, based on the extent of necrotic areas of the aerial parts of the plants. The scale ranged from 0 to 5 where 0 represented no damage and 5 a dead plant. Frost damages observations were realized at four dates in 2008: January, 4th and 15th, March, 28th and April, 10th.

A frost experiment was also conducted in a controlled environment chamber using the standardized test described previously by Dumont et al. [22], which mimics the successive periods of cold acclimation and frost generally encountered in the field by autumn-sown peas. Pea accessions were placed according to a randomized complete block design with three replicates. To provide three biological replicates, the experiment was carried out three times successively in the same controlled environment chamber. The temperature, light level and humidity were recorded and were similar during the three experiments. Briefly, the plants at the stage of 2nd - 3rd leaf were first treated with a regime of 11 days of cold acclimation at 10 °C/2 °C (day/night) with a 10 h photoperiod. The frost treatment was then carried out at 6 °C/− 8 °C with 8 h of daylight during 4 days. After frost, a recovery period was applied with a temperature regime of 16 °C/5 °C and 10 h of daylight during 8 days. Frost tolerance was evaluated by scoring frost damages at the end of the recovery period with the same scoring scale as the one used to evaluate the frost damages in the field experiment, except that scores was attributed to single plants instead of plots.

Overall, 5 traits constituted the phenotyping data for the GWAS, abbreviated as follows: FD_CC: frost damages in the controlled conditions experiment, FD_Field_date1, FD_Field_date2, FD_Field_date3 and FD_Field_date4: winter frost damages evaluated in the field experiment at the first, second, third and fourth date respectively.

### Phenotypic data analyses

The phenotypic data were analyzed with the R 3.5.0 software [56, 57] using a linear mixed model to obtain estimates of variance components, heritability (H^2^), as well as best linear unbiased predictions (BLUPs) of adjusted means. The following Linear Mixed Model (LMM) was used: Y_ij_ = μ + geno_i_ + rep_j_ + e_ij_, where Y_ij_ is the value of frost damages recorded for the genotype *i* at the replicate *j*. μ is the mean, geno_*i*_ is the random genetic effect of the genotype *i*, rep_*j*_ is the fixed replicate effect of the replicate *j* and e_*ij*_ is the residual effect. The model was carried out using the R function “lmer” of the package ‘lme4’ [58, 59]. Heritability (H^2^) was estimated using the following formula: $$ {\mathrm{H}}^2={\mathrm{V}}_{\mathrm{g}}/\left({\mathrm{V}}_{\mathrm{g}}+\raisebox{1ex}{${\mathrm{V}}_{\mathrm{res}}$}\!\left/ \!\raisebox{-1ex}{${\mathrm{n}}_{\mathrm{rep}}$}\right.\right) $$, where V_g_ is the genotypic variance component, V_res_ is the residual variance component and n_rep_ is the number of replicates taking into account the missing values. BLUPs for each genotype-trait combination were calculated from each LMM analysis using the function “ranef”, implemented in ‘lme4’ package of R software [59] and were used for the GWA analysis.

### Genotyping and quality control

The collection was genotyped at 11,366 SNPs using the Illumina Infinium® BeadChip 13.2 K SNPs as described in [36]. These SNPs were all located in gene-context sequences and derived from separated transcripts [60]. The consensus genetic map from Tayeh et al. [36] was used as the genetic framework for the association analyses. This map was built on the basis of genotyping data collected for 12 pea recombinant inbred line populations. Considering the large collinearity between individual maps, a set of genotyping data for 15,352 markers and from all populations was used to build the consensus map. The latter shows a cumulative total length of 794.9 cM and a mean inter-marker distance of 0.24 cM.

The genotyping matrix, which was composed of a set of 11,366 SNPs and 365 pea accessions, was filtered using Plink v.1.9 software [61, 62]. Accessions and SNP markers with a call rate below 0.90 as well as SNP markers with a minor allele frequency (MAF) below 0.05 were excluded from the GWA analysis. After quality control checking, a genotyping matrix consisting of 10,739 SNPs and 363 accessions with 0.6% missing data was kept for further analyses. The resulting data set was further imputed using Beagle v.3.3.2 software [63]. Beagle applies a Markov model to the hidden states (the haplotype phase and the true genotype) along the chromosome using an EM (Expectation-Maximization) algorithm that iteratively updates model parameters to maximize the model likelihood up to the moment where convergence is achieved. Finally, a genotyping matrix consisting of 10,739 SNPs and 363 accessions with no missing data was used for GWAS. Scripts from Negro et al. [64] were used for the quality control and imputation.

### Linkage disequilibrium estimation

The estimates of linkage disequilibrium (LD) within the collection were determined by the squared allele-frequency correlations (*r*^2^) for pairs of loci as described in Weir [65]. Linkage disequilibrium analysis between pairs of SNP markers was calculated in a sliding window of 900 markers using Plink v.1.9 software [61, 62]. Then, intrachromosomal LD quantification and graphical representation of LD decay were accomplished using R 3.5.0 software [56, 57]. The LD decay was measured as the genetic distance (cM) where the average *r*^2^ decreased to half its maximum value.

### Population structure and individual relatedness

To control false positive associations, population structure and individual relatedness (kinship) among accessions of the collection were taken into account by fitting markers based structure and kinship matrices in the association models [66]. Kinship and population structure were estimated using a matrix data composed of 363 accessions and a set of 2962 markers without any missing data and corresponding to non-redundant genetic positions randomly selected on the consensus map. The coefficients of kinship between pairs of accessions were estimated using the realized relationship matrix kinship estimation approach implemented in FaST-LMM software [67]. Two alternative approaches were considered to estimate the kinship matrix as described by Rincent et al. [68]. In the first one, the kinship was estimated with all the markers that are not located on the same linkage group (LG) than the tested SNP. Thus, seven kinship matrices were estimated, each being specific to a linkage group; these matrices were noted K_LGx_ with x corresponding to the number of linkage group tested. Such an approach aims at increasing power of detection of significant markers in GWAS particularly in regions of high LD. In the second approach, correlation between markers took into account all the 2962 markers and the kinship matrix was noted K.

The discriminant analysis of principal components (DAPC) method developed by Jombard et al. [69] and implemented into the ‘adegenet’ R package [70–72] was used to cluster accessions on the basis of their genotype. This method aims at identifying and describing clusters of genetically related individuals without prior knowledges of groups. First, the optimal number of genetic clusters (k) was determined through the ‘K-means’ method using the function “find.clusters”. The number of clusters was allowed to vary from one to 20 during the determination of the optimal value of k, based on the Bayesian Information Criterion (BIC). The most likely number of clusters was chosen on the basis of the lowest associated BIC. Then, the principal component analysis (PCA) step of DAPC was performed through maximization of the ‘a-score’ criterion and the optimal number of principal components (PCs) was obtained after 100 iterations using the function “optim.a.score” implemented in ‘adegenet’ package of R software. Finally, DAPC was performed considering the most likely number of clusters (k) and the optimal number of PCs identified using the function “dapc” implemented in adegenet R-package [70–72]. To confirm the allocation of accessions to clusters by DAPC analysis, a Nei genetic distance matrix [73] was calculated with the function “stamppNeisD” implemented in ‘StAMPP’ package of R software [74] using the genotyping data composed of 363 accessions and a set of 2962 SNPs. Then the resulting matrix was plotted as a dendrogram using the ward method with the package ‘cluster’ implemented in R software [75].

The two first coordinates of DAPC results were used as covariates (Q matrix) in the GWAS to correct the association tests for false positives.

### Association mapping

BLUPs corresponding to the phenotypic data collected for each accession were used to identify marker-trait association using Linear Mixed Model (LMM) accounting for kinship matrix (K or K_LG_) with or without population structure matrix (Q) as random effect(s). Four models were therefore compared for their capacity to fit the data: (1) a LMM using the kinship matrix K, (2) a LMM corrected for kinship using the K_LG_ matrices, (3) a LMM including the K and Q matrices and (4) a LMM using both K_LG_ and Q matrices. For each frost damages trait, the best model was chosen by comparing the likelihoods of each model using the Bayesian Information Criterion (BIC) [76]. The model with the smallest BIC was selected. All analyses were performed using LMM provided by the FaST-LMM version algorithm [67]. The threshold to declare an association significant was set at a probability level of the *p*-value inferior to 4.65E-06, i.e. -log10 (*p*) > 5.33, which corresponded to the Bonferroni threshold (0.05/ number of tested SNPs). To represent the association results, Manhattan plots and their corresponding quantile-quantile plots were drawn using the package ‘QQman’ implemented in R software [77].

### Local LD block estimation and favorable allele identification

Local LD analysis was used to define the LD blocks around significant associated markers detected by GWAS using Plink 1.9 software [61, 62]. For each associated marker, markers in strong linkage disequilibrium (LD; *r*^2^ > 0.8) with this one, were identified to define a LD block. By this way, a LD block was defined as the interval including all markers in LD (*r*^2^ > 0.8) with the targeted associated marker(s). Unique associated markers which didn’t constitute a LD block were kept for further analyses. Thus, for each identified genomic region, LD blocks and unique associated marker(s) composed a significant locus related with frost tolerance. At each significant locus, haplotypes were identified, among the accessions of the collection, according to the non-imputed genotyping data corresponding to the list of markers significantly detected by GWAS and linked markers. Haplotypes showing missing data loci as well as SNP with heterozygous genotypic data were excluded from further analysis. Besides, haplotypes represented by less than 5% of the total number of accessions were also removed from the analysis. Based on the results of association mapping, the allelic effect corresponding to the minor allele (a_eff_) of markers significantly associated with the frost damage traits were analyzed: if a_eff_ had a negative value, the minor allele of the associated marker was considered to decrease frost damage (favorable allele for frost tolerance); if a_eff_ had a positive value, the minor allele of the associated marker was considered to increase frost damage (unfavorable allele for frost tolerance). For each significant locus and each corresponding trait, the values of frost damages of the different haplotypes were compared using an analysis of variance with a nested design for ‘haplotype/genotype’, followed by a Student-Newman-Keuls (SNK) comparison test using the function “SNK.test” of the R-package ‘agricolae’ [78]. Favorable and unfavorable haplotypes at each significant locus were defined as follows: the favorable haplotypes should show a significant lower frost damage mean score while unfavorable haplotypes should show a significant higher frost damage mean score. Finally, we listed representative accessions for each favorable and unfavorable haplotype based on the following condition: each accession should show a mean score of the considered associated trait(s) inferior to 1 for favorable haplotypes and superior to 4 for unfavorable ones.

### Annotated genes underlying frost tolerance loci

To identify genes that may be associated with the frost damage phenotypes, a region encompassing 1 Mb flanking regions upstream and downstream from each of the FD-related markers, ie. significant GWAS markers and markers in LD (*r*^2^ > 0.8) with the former ones, was defined. This region was searched for genes annotated in the pea genome assembly v.1a developed by Kreplak et al. [39] using the genome JBrowse available at https://urgi.versailles.inra.fr/Species/Pisum.

## Supplementary information

**Additional file 1: Figure S1.** Distribution of Best Linear Unbiased Prediction (BLUP) values for the five traits observed within the pea collection. A: frost damages in the controlled conditions experiment. B, C, D and E: frost damages in the field experiment at the date 1, 2, 3 and 4 respectively.

**Additional file 2: Figure S2.** Distribution of 10,739 SNPs along the *Pisum sativum* linkage groups. Number of SNPs per position are indicated as grey horizontal bars. Genetic position in cM is shown on the y-axis and number of SNPs per position is shown on the x-axis.

**Additional file 3: Table S1.** Description of the *Pisum sativum* linkage groups (LGs) used in the present study. The number of SNP markers, the genetic length (in cM, from Tayeh et al. [36]) and the average minor allele frequency (MAF) are shown for each LG.

**Additional file 4: Figure S3.** Distribution of minor allele frequencies (MAF) for 10,739 SNP markers within the 363 pea accessions.

**Additional file 5: Figure S4.** Scatterplot showing the linkage disequilibrium (LD) decay estimated in the association mapping collection. The LD decay across each linkage group (LG) and the overall LD decay across the genome (All LG) are shown. The *r*^2^ values of LD between pairs of markers considered are plotted as a function of the genetic position in cM. Red curves represent the estimated LD decay. Blue dashed horizontal lines represent half of the maximum LD value. Blue dashed vertical lines represent the estimated genetic distance (cM) at which the LD decay dropped to half of its maximum. LD decay rate is represented as the point of intersection between the two dashed lines.

**Additional file 6: Table S2.** Description of the association mapping collection. This table presents the list of the pea accessions composing the association mapping collection with their end-use, cultivation status, geographical origin and sowing type. The ‘DAPC_Cluster (k)’ column shows assignation of the 363 pea accessions to a cluster based on the discriminant analysis of principal components (DAPC). The ‘Dendrogram_Cluster (k)’ column shows the allocation of individuals to clusters based on the dendrogram using Nei genetic distances between accessions. The description of the pea accessions is extracted from Burstin et al. [55]. CRB Code: Code used for the association mapping, also named collection of biological resources (CRB). *: sowing type modified for this accession, according to Yenne et al. [79].

**Additional file 7: Figure S5.** Dendrogram from Nei genetic distance matrix for 363 genotypes of the pea reference collection. On the y-axis are represented the genetic distances between clusters or accessions. On the x-axis are represented, in red font, the clusters identified for a Nei genetic distance of 7.

**Additional file 8: Figure S6.** Distribution of the kinship coefficients between accessions of the association mapping collection. The first histogram (A) describes the distribution of the kinship coefficients within the K matrix, calculated with all markers of the genome. The remaining histograms (B, C, D, E, F, G and H) describe the kinship coefficients within each of the seven K_LG_ matrices calculated as explained in the material and methods section (for example the kinship matrix K_LG1_ was estimated with all the markers except those that are located on the first linkage group).

**Additional file 9: Table S3.** Description of linkage disequilibrium (LD) blocks per linkage group in the association mapping collection. A LD block consists in a series of at least 2 markers which are in significant LD (*r*^2^ > 0.8) with at least one trait-associated marker (underlined marker). LD blocks are named in consecutive numerical order following their linkage group (LG) name. cM*: genetic position, in centiMorgan of each marker along the genetic map of the corresponding linkage group; LD (*r*^2^) **: *r*^2^ value of each marker with the other markers of the same LD block.

**Additional file 10: Table S4.** Marker haplotype analysis of the association mapping collection. For each linkage group (LG), the list of markers significantly detected by GWAS and markers in linkage disequilibrium (LD; *r*^2^ > 0.8) with the former ones is shown. The third line shows genetic positions from the consensus map of Tayeh et al. [36]. The fourth line indicates the LD blocks composed and named as mentioned in the legend of Additional file 9. The following lines show the allelic composition of haplotypes defined by LD blocks and individual associated markers at each of the 6 frost tolerance loci on linkage groups (LGs) I, II, III, V, VI and VII. For each frost damage (FD)-associated marker, the favorable allele is in red font and the unfavorable allele in blue font. Haplotypes are named in consecutive numerical order following their linkage group name; only haplotypes without missing values or heterozygous markers and carried by more than 3% of the lines from the association mapping collection are listed. For each haplotype, accessions and their mean phenotypic values ± standard error of the variables significantly associated with marker(s) in the linkage group are shown. Significant differences between haplotypes were assayed by a SNK means comparison test; favorable haplotypes are shown by a red background and unfavorable haplotypes by a blue background, regarding the SNK test. Haplotypes with a white background are classified in intermediate groups.

**Additional file 11: Table S5.** List of annotated genes underlying genome-wide association loci of frost tolerance in pea. Genes that were located in an interval of ±1 Mb on both sides of markers significantly detected by GWAS and markers in linkage disequilibrium (LD; *r*^2^ > 0.8) with the former ones, are listed. For each identified gene, the nearest marker significantly detected by GWAS (underlined font) or marker in LD with associated marker(s) (non-underlined font) is shown. The annotation of genes was extracted from *Pisum sativum* v.1a genome JBrowse available at https://urgi.versailles.inra.fr/Species/Pisum [39]. Genes positions in the pea genome assembly v.1a are presented by their assigned chromosomes and physical positions indicated in bp. Genes which are not assigned to one of the seven chromosomes, are represented by their physical positions on unanchored scaffolds. ^a^: Annotation refined with the homologous gene from *Medicago truncatula* available on the *Pisum sativum* v.1a genome JBrowse, and whose corresponding gene function was identified from the *Medicago truncatula* v4.0 genome JBrowse available on www.medicagogenome.org. ^b^: Annotation refined with the homologous gene from *Glycine max* available on the *Pisum sativum* v.1a genome JBrowse and whose corresponding gene function was identified from the genome v9.0 assembly V1.1 available on http://soykb.org/gene_card.php. ^c^: Annotation refined with the predicted protein function of transcript sequences corresponding to mapped SNPs, extracted from Table S10 in Tayeh et al. [36].

**Additional file 12: Table S6.** Description of the pea association mapping collection as described in Additional file 6 and the correspondence of the genotyping results of accessions at the *Hr* locus.

**Additional file 13: Table S7.** Description of accessions of the pea reference collection for their haplotype at the frost damage (FD)-associated loci of the GWA study and at the *Hr* locus. At the linkage groups (LGs) I, II, III, V, VI and VII, the favorable haplotypes are shown by a red background, the unfavorable haplotypes by a blue background, as described in the legend of Additional file 10. Accessions with undefined haplotypes or intermediate haplotypes were not presented. The same colour code has been used to describe the favorable (red background: Hr) and unfavorable (blue background: hr) allele for the *Hr* gene, as determined in Lejeune-Hénaut et al. [21]. The mean frost damage score observed in the field experiment as well as its standard error (SE) are given. Frost scores are ranging from 0 (no damage) to 5 (dead plant). The passport data of accessions are extracted from the Additional file 6.

**Additional file 14: Table S8.** Daily air and soil temperatures during the field experiment in Clermont-Ferrand Theix.

## Data Availability

Genetic positions and sequences of markers used for the current study are available in Tayeh et al. [36].The pea genome assembly v.1a explored in this study is available at https://urgi.versailles.inra.fr/Species/Pisum (Kreplak et al. [39]). The data supporting the findings of this study were used under license within the PeaMUST project and are not publicly available. Phenotyping and genotyping data, all other intermediate data files and scripts are however available from the authors upon reasonable request and subjected to data transfer agreement.

## Abbreviations

AP2/EREBP: APETALA2/Ethylene Responsive Element Binding Protein
BIC: Bayesian Information Criterion
BLUPs: Best Linear Unbiased Predictions
CBF: C-repeat Binding Factor
cM: centi Morgan
COR: COld Regulated
CVg: Coefficient of Variation of the genetic variance
DAPC: Discriminant Analysis of Principal Components
DREB: Dehydration-Responsive Element-Binding
Elf3: Early flowering 3
FD: Frost Damage
FD_CC: Frost damages in the controlled conditions experiment
FD_Field_date1: Frost damages in the field experiment at the first date of scoring
FD_Field_date2: Frost damages in the field experiment at the second date of scoring
FD_Field_date3: Frost damages in the field experiment at the third date of scoring
FD_Field_date4: Frost damages in the field experiment at the fourth date of scoring
GWAS: Genome Wide Association Study
H^2^: Broad sense heritability
Hr: High response to photoperiod
INRAE: National Research Institute for Agriculture, Food and Environment
k: DAPC Cluster
K: Kinship matrix
K_LG_: Kinship matrix corresponding to a given Linkage Group
LD: Linkage Disequilibrium
LG: Linkage Group
LMM: Linear Mixed Model
MAF: Minor Allele Frequency
NAM: Nested Association Mapping
NIL: Near Isogenic Line
PCs: Principal Components
PCA: Principal Components Analysis
Q: Structure matrix
QTL: Quantitative Trait Locus
RIL: Recombinant Inbred Line
SE: Standard Error
SNK: Student-Newman-Keuls
SNP: Single Nucleotide Polymorphism
SSD: Single Seed Descent
SSR: Simple Sequence Repeat
Vg: Genetic variance
WFD: Winter Frost Damage
