# Supplementary figures and images for: Genome-wide association study identifies favorable SNP alleles and candidate genes for frost tolerance in pea
Source: BMC Genomics. 2020 Aug 4;21:536. doi: 10.1186/s12864-020-06928-w (PMC7430820; doi:10.1186/s12864-020-06928-w)

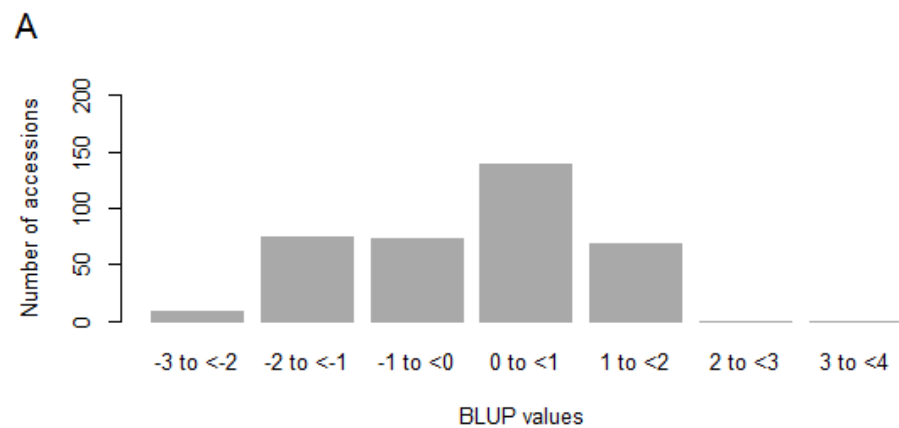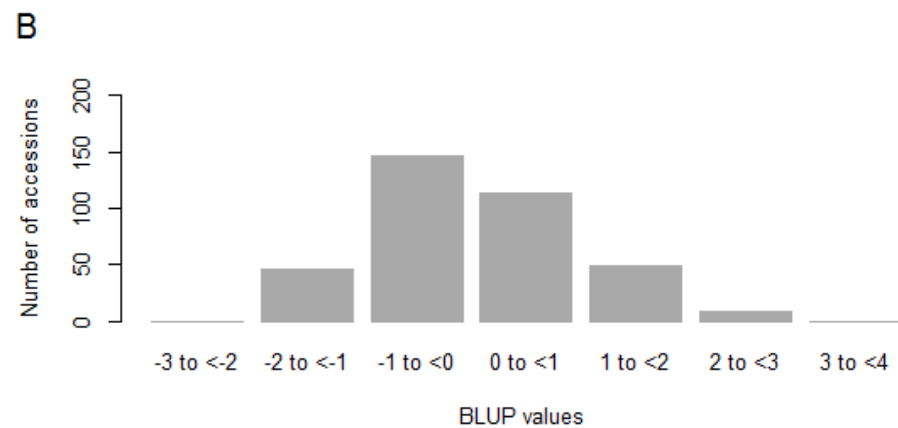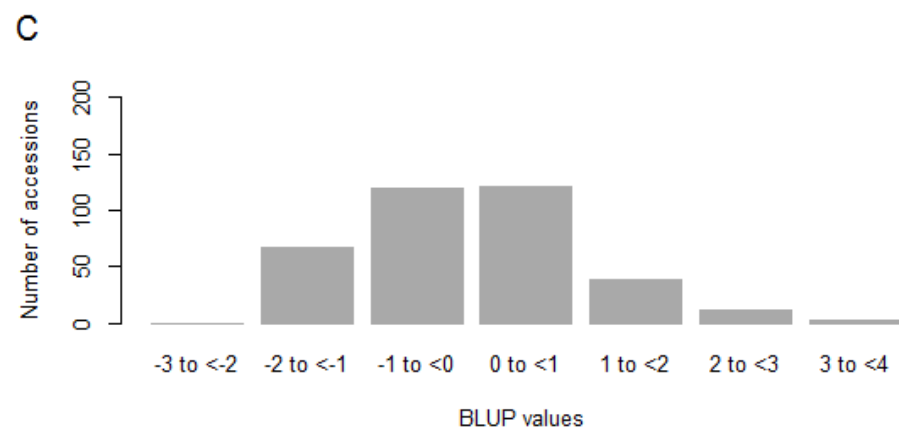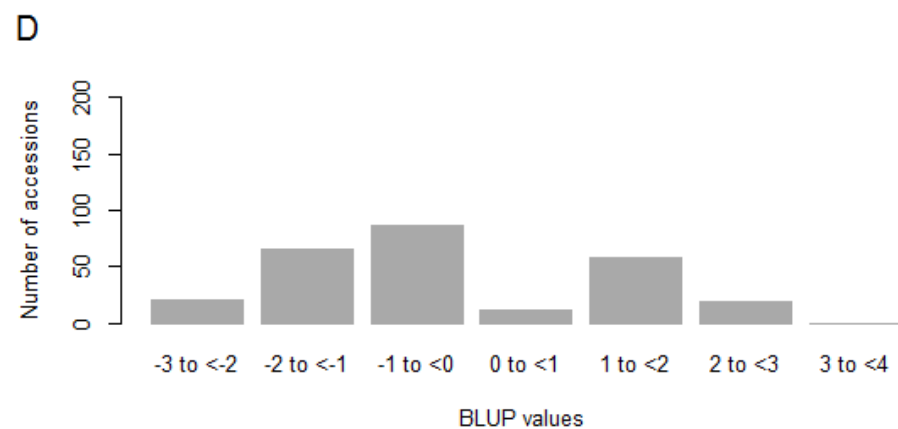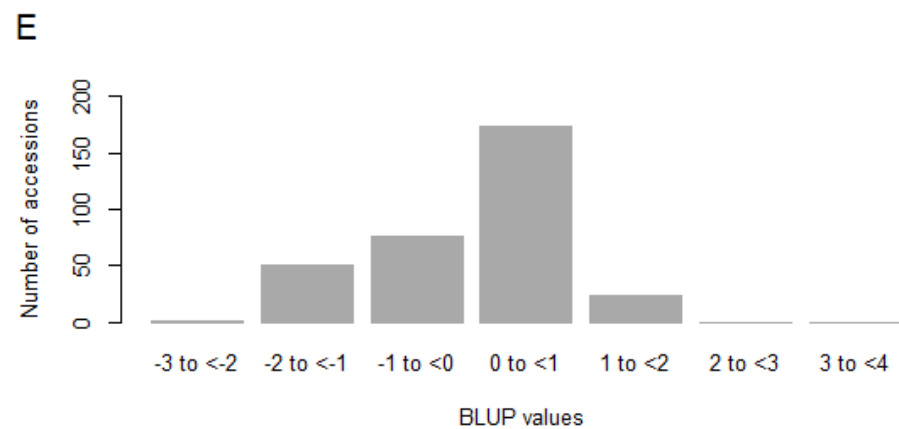

Supplement: Supplementary file 1 — Additional file 1: Figure S1. Distribution of Best Linear Unbiased Prediction (BLUP) values for the five traits observed within the pea collection. A: frost damages in the controlled conditions experiment. B, C, D and E: frost damages in the field experiment at the date 1, 2, 3 and 4 respectively. [file 12864_2020_6928_MOESM1_ESM.pdf]

LGI

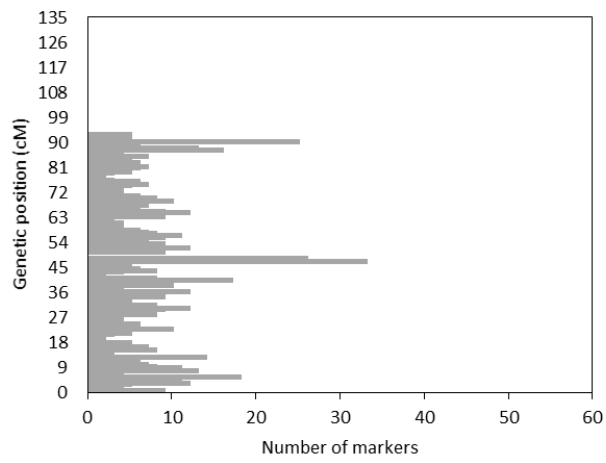

LGII

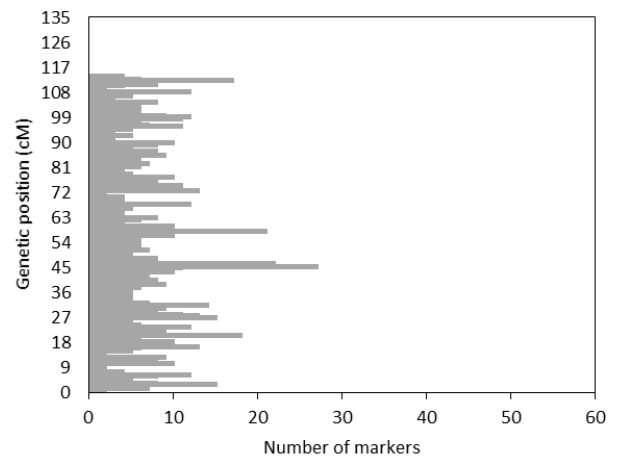

LGIII

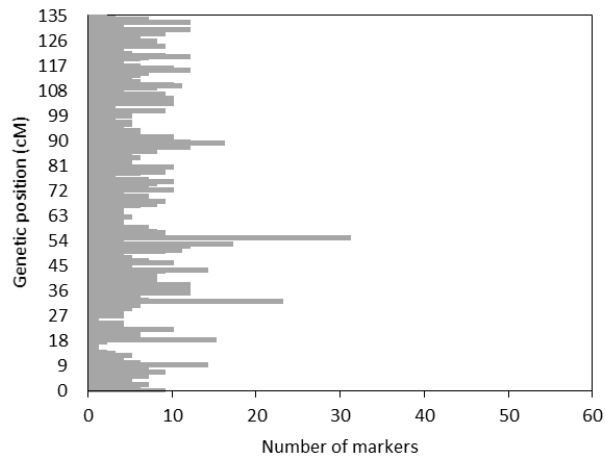

LGIV

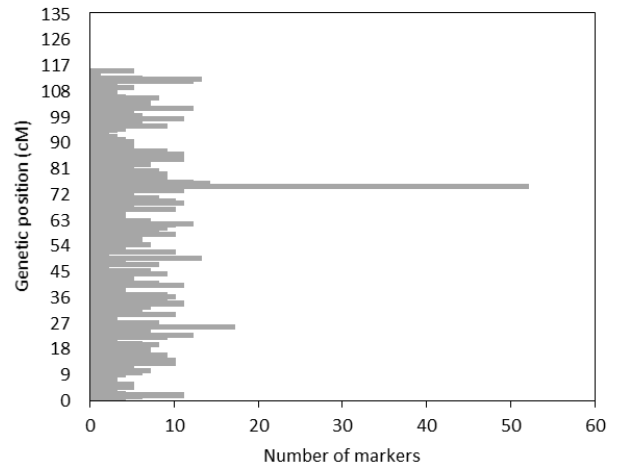

LGV

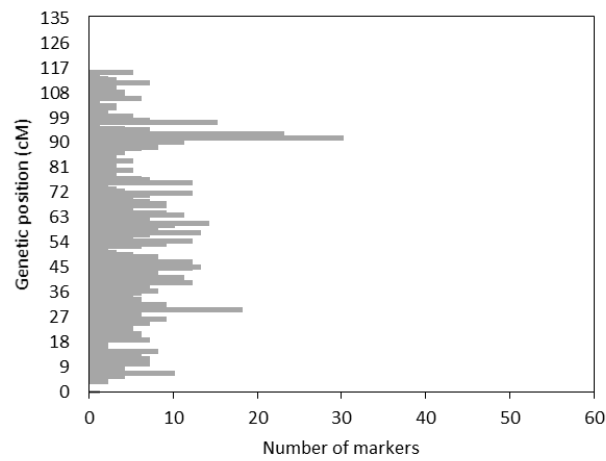

LGVII

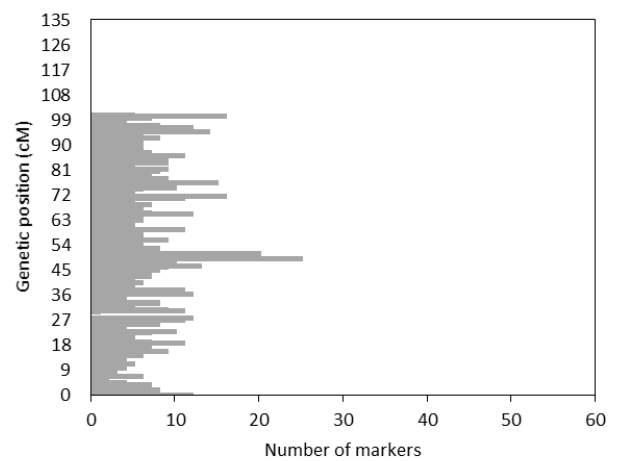

LGVII

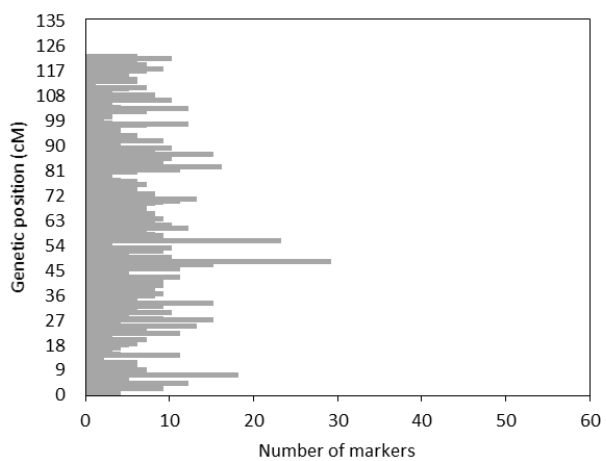

Supplement: Supplementary file 2 — Additional file 2: Figure S2. Distribution of 10,739 SNPs along the Pisum sativum linkage groups. Number of SNPs per position are indicated as grey horizontal bars. Genetic position in cM is shown on the y-axis and number of SNPs per position is shown on the x-axis. [file 12864_2020_6928_MOESM2_ESM.pdf]

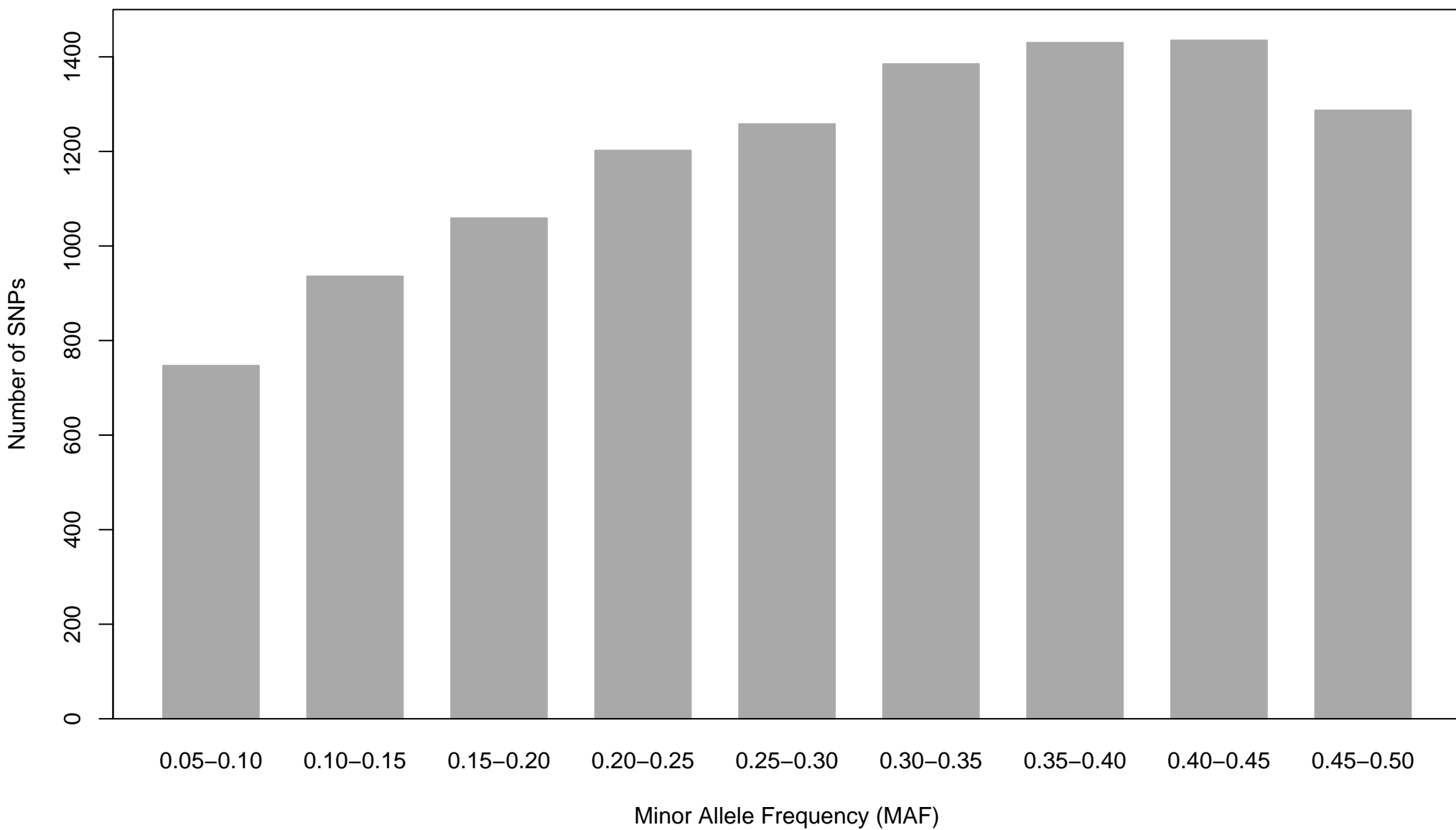

Supplement: Supplementary file 4 — Additional file 4: Figure S3. Distribution of minor allele frequencies (MAF) for 10,739 SNP markers within the 363 pea accessions. [file 12864_2020_6928_MOESM4_ESM.pdf]

**LG I**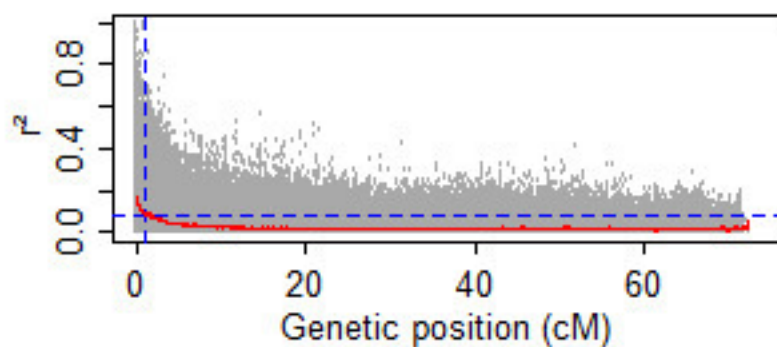**LG II**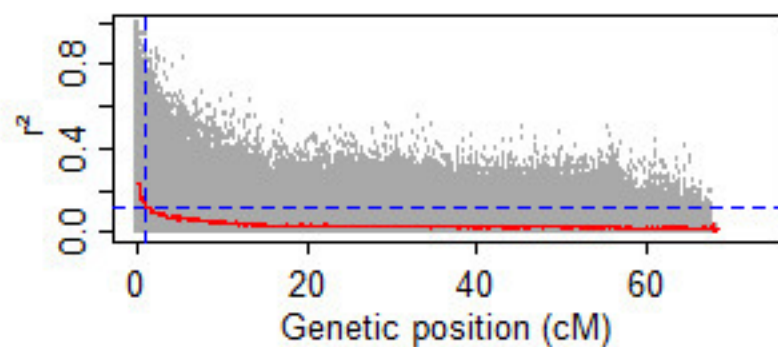**LG III**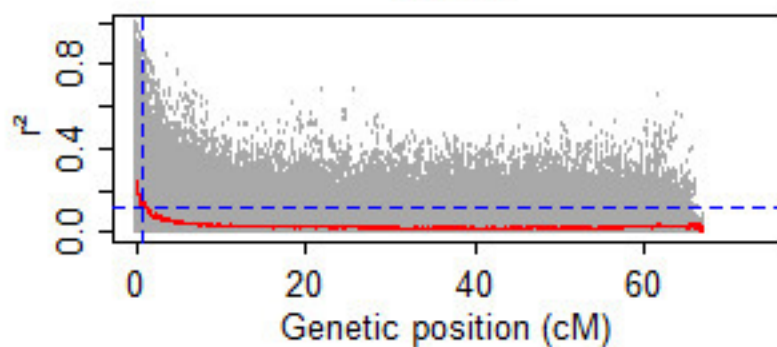**LG IV**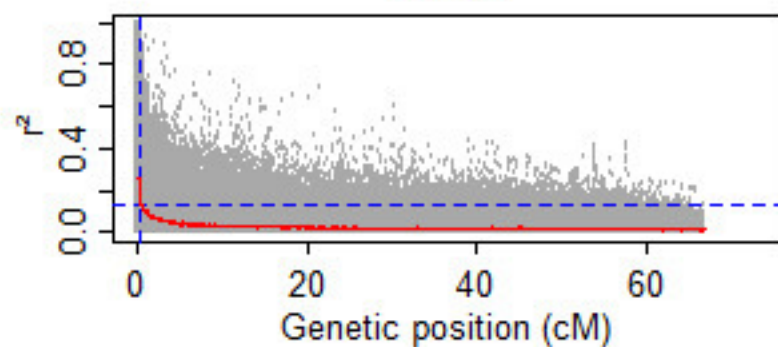**LG V**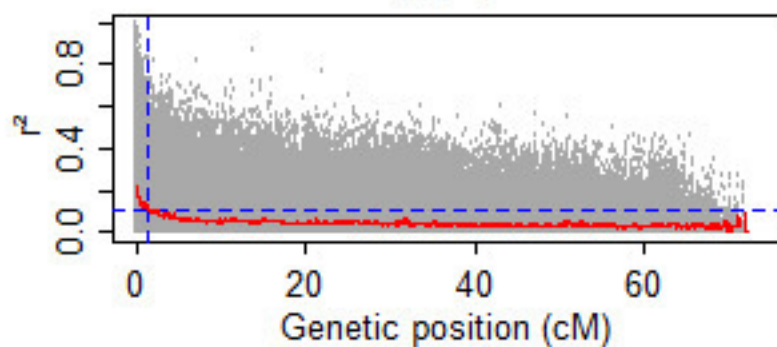**LG VI**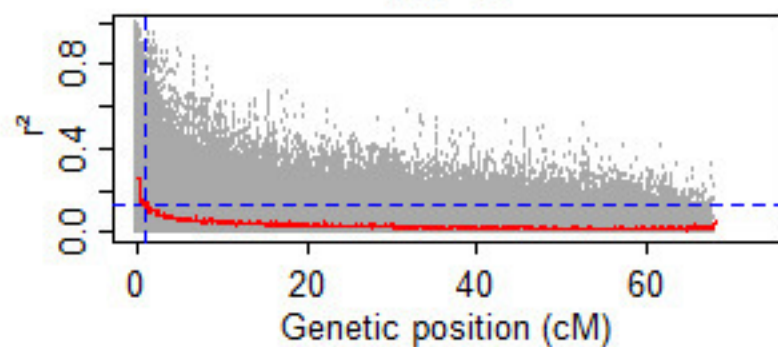**LG VII**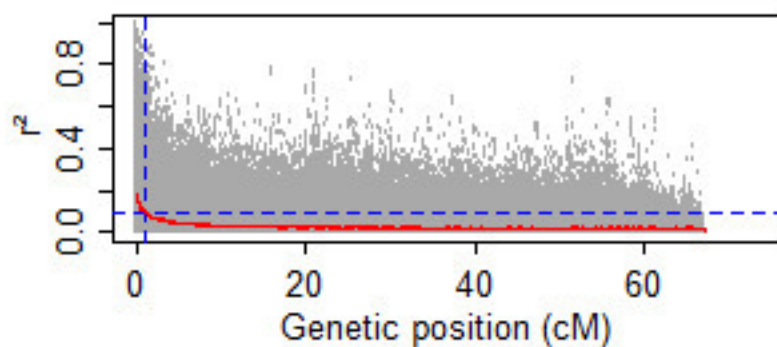**All LG**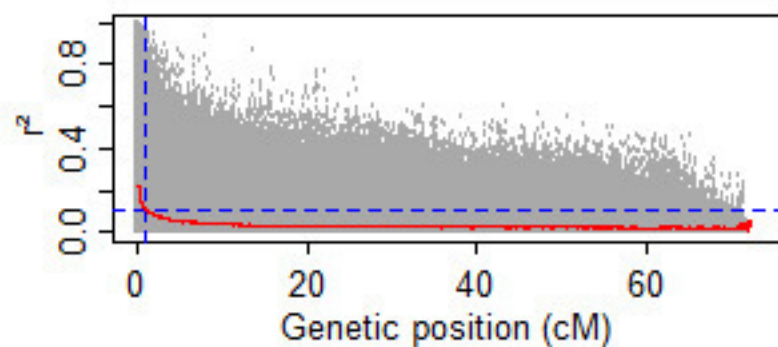

Supplement: Supplementary file 5 — Additional file 5: Figure S4. Scatterplot showing the linkage disequilibrium (LD) decay estimated in the association mapping collection. The LD decay across each linkage group (LG) and the overall LD decay across the genome (All LG) are shown. The r2 values of LD between pairs of markers considered are plotted as a function of the genetic position in cM. Red curves represent the estimated LD decay. Blue dashed horizontal lines represent half of the maximum LD value. Blue dashed vertical lines represent the estimated genetic distance (cM) at which the LD decay dropped to half of its maximum. LD decay rate is represented as the point of intersection between the two dashed lines. [file 12864_2020_6928_MOESM5_ESM.pdf]

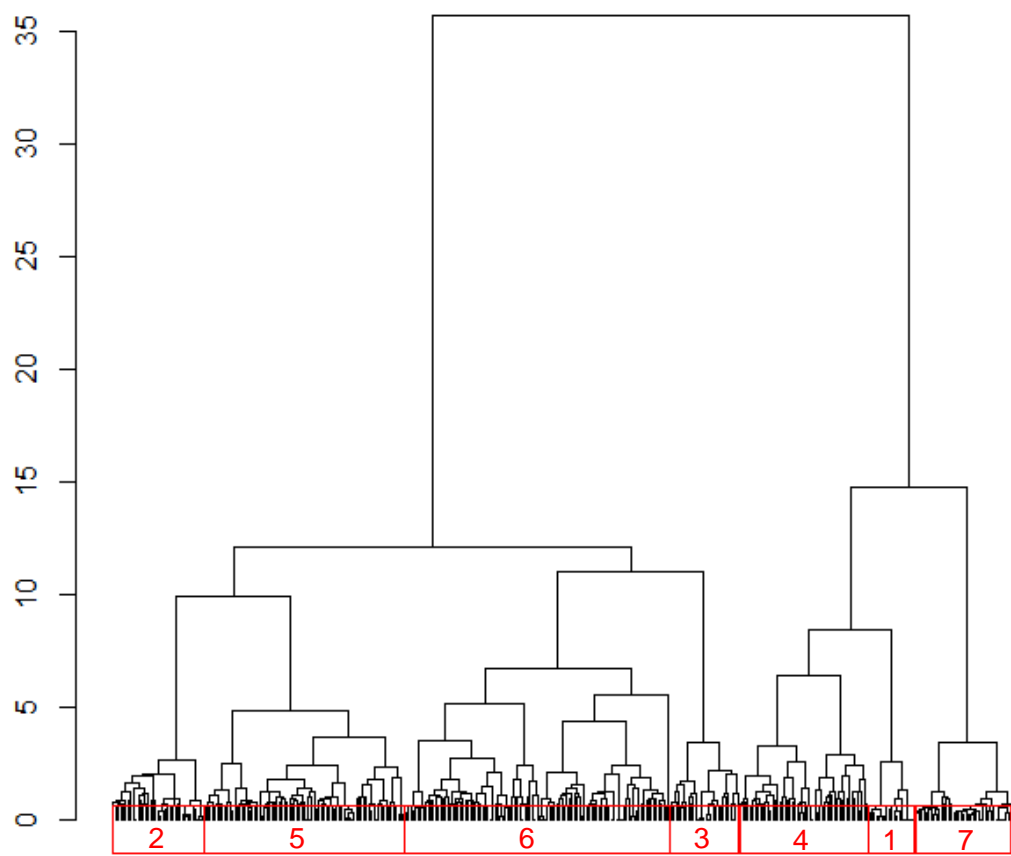

Supplement: Supplementary file 7 — Additional file 7: Figure S5. Dendrogram from Nei genetic distance matrix for 363 genotypes of the pea reference collection. On the y-axis are represented the genetic distances between clusters or accessions. On the x-axis are represented, in red font, the clusters identified for a Nei genetic distance of 7. [file 12864_2020_6928_MOESM7_ESM.pdf]

**A**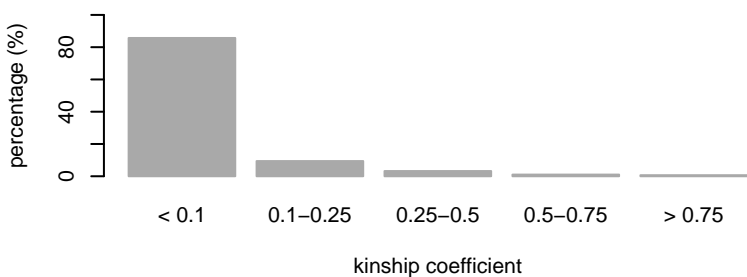**B**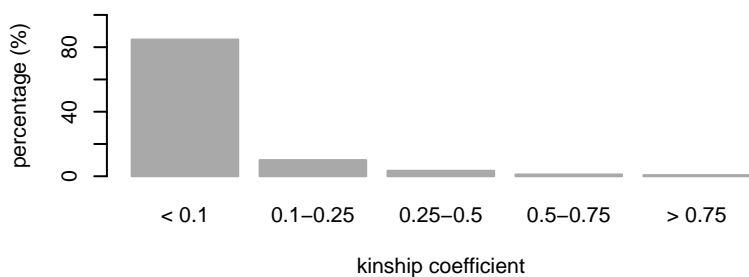**C**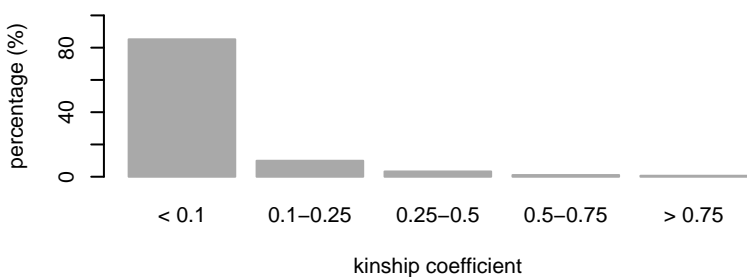**D**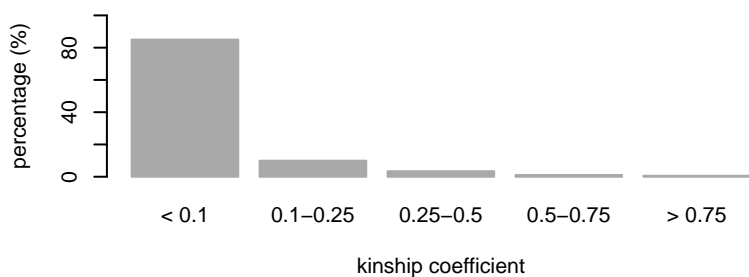**E**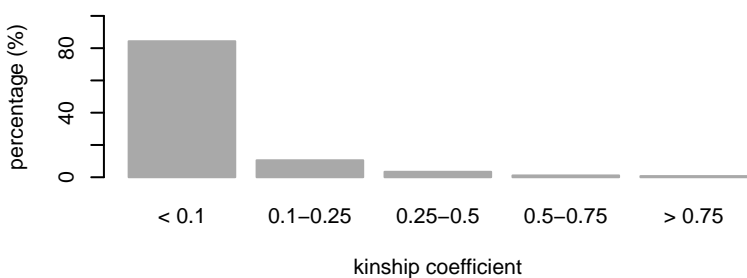**F**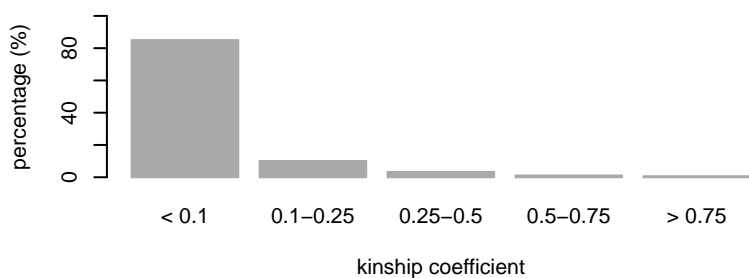**G**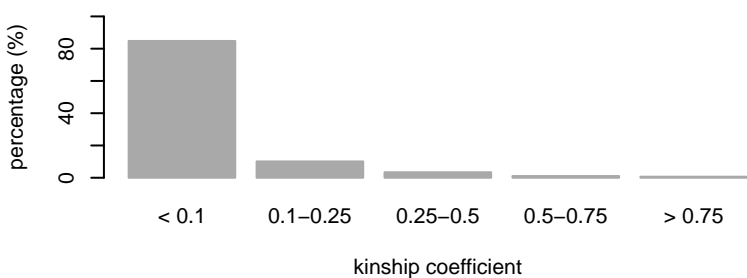**H**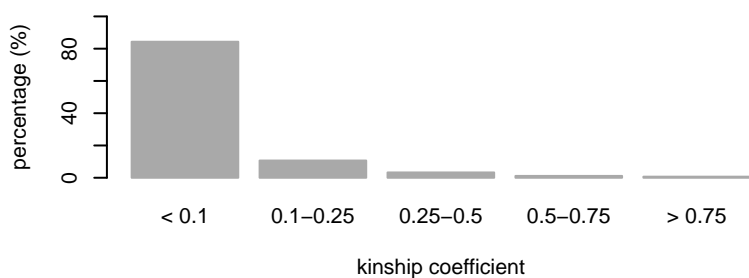

Supplement: Supplementary file 8 — Additional file 8: Figure S6. Distribution of the kinship coefficients between accessions of the association mapping collection. The first histogram (A) describes the distribution of the kinship coefficients within the K matrix, calculated with all markers of the genome. The remaining histograms (B, C, D, E, F, G and H) describe the kinship coefficients within each of the seven KLG matrices calculated as explained in the material and methods section (for example the kinship matrix KLG1 was estimated with all the markers except those that are located on the first linkage group). [file 12864_2020_6928_MOESM8_ESM.pdf]
